# High-Efficiency l‑PEI-Based Transfection of ARPE-19 Cells Using a Multiparametric Approach and Automated Polyplex Formation with a 3D-Printed Microfluidic System

**DOI:** 10.1021/cbe.5c00059

**Published:** 2025-09-10

**Authors:** Daniel Keim, Michaela Dehne, Patricia Miller, Valérie Jérôme, Janina Bahnemann, Ruth Freitag

**Affiliations:** † Process Biotechnology, 26523 University of Bayreuth, Universitätstraße 30, Bayreuth 95444, Germany; ‡ Technical Biology, Institute of Physics, 26522 University of Augsburg, Universitätsstraße. 1, Augsburg 86159, Germany; § Institute of Technical Chemistry, Leibniz University Hannover, Callinstraße 5, Hannover 30167, Germany; ∥ Centre for Advanced Analytics and Predictive Sciences (CAAPS), University of Augsburg, Universitätsstraße 6, Augsburg 86159, Germany

**Keywords:** nonviral gene delivery, human retinal pigmented epithelia cells, polycation, l-PEI, microfluidic, 3D printing

## Abstract

Nonviral gene delivery offers promise for treating age-related macular degeneration (AMD), a major cause of blindness. Genetic modification of retinal pigment epithelium (RPE) cells is a potential therapeutic strategy for AMD. This study presents a multiparametric approach to enhance nonviral transfection of human ARPE-19 cells using linear poly­(ethylenimine) (l-PEI, 25 kDa) as a delivery agent for plasmid DNA (pDNA). The transfection protocol was optimized by adjusting the N/P ratio through nucleic acid concentration, varying polymer density, reducing transfection volume, and minimizing contact time between cells and polyplexes. Under optimized conditions, transfection efficiency (TE) reached 88% with ∼85% viability. A semi-automated method for polyplex formation was developed using a 3D-printed microfluidic system, thereby enabling standardized production. This optimized protocol was successfully adapted to the microfluidic system without compromising TE or viability. This semi-automated approach represents a step toward the scalable and reproducible application of l-PEI-based transfection technologies for future therapeutic use.

## Graphical abstract

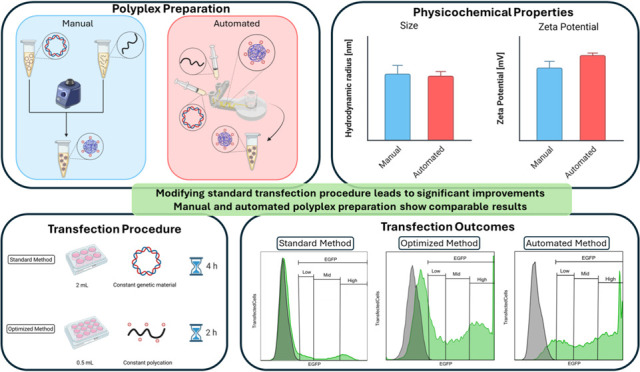

## Introduction

Age-related macular degeneration (AMD) is one of the most common causes of irreversible blindness in the elderly.^1,2^ Approximately 200 million people worldwide are affected by some form of AMD, which is classified into two types: “dry” (atrophic) and “wet” (neovascular).^3^ While treatment is now available for the “wet” form, this is unfortunately still not the case for the more prevalent “dry” form.^1–4^ DNA-based ocular gene therapy (e.g., expression of the complement factor I (GT-005) and protein CD59 (HMR59)—both Adeno-Associated Virus (AAV)-mediated approaches) has shown promise in clinical trials for treating “dry” AMD.^5,6^ Additionally, Devoldere et al. have proposed chemically stabilized mRNA as therapeutic alternative for retinal diseases.^7^ Such strategies involve the genetic modification of retinal pigment epithelial (RPE) cells to express a specific recombinant protein (e.g., complement factor I, CD59),^5,6^ thereby ensuring cell survival and maintaining functionality.^1^ While viral vectors are easy to load with genetic material and achieve high transfection efficiencies (TE), significant drawbacks remain - including size-restrictions of the genetic cargo, immunogenicity, and potential toxicity.^8–10^ Nonviral vectors, such as polymers or lipids can transport larger genetic payload, have a reduced immunogenicity, and are also highly adjustable (i.e., design flexibility and functionalization).^11–13^ Yet, the TE of nonviral vectors is generally lower than their viral counterparts, especially for end-differentiated and nondividing primary cells, and their cytotoxicity still remains a cause for concern.^14^ Delivering genetic material to RPE cells using nonviral transfection agents continues to post significant challenges due to these confounding factors.

The ARPE-19 cell line (spontaneously arisen from human RPE cells) is a widely used model due to its retention of certain key RPE characteristics, including monolayer growth, cobblestone morphology, and the expression of markers like cellular retinaldehyde-binding protein (CRALBP) and RPE-65.^15^ These features make ARPE-19 cells a suitable model for developing transfection methods for retinal gene therapy and to date several nonviral transfection strategies have been explored with respect to these cells, albeit only with moderate success.^16–18^ Physical methods (such as nucleofection) have thus far achieved the best results with a TE of ≈80% but these methods are impractical for *in vivo* applications.^19^ Chemical methods (such as Lipofectamine) have also shown promising results with TE up to 60%.^20,21^ Polycation-based strategies have similarly demonstrated some success; for example, diacrylate-based polymers have achieved 44% TE with 77% viability,^18^ while dendrimeric-lipid formulations have reached up to 80% TE and high viability in serum-containing media.^22^ And notably, branched poly­(ester amine) polymers have also demonstrated an ability to facilitate pDNA delivery into RPE cells.^23^ But most of the proposed systems rely on complex formulations or even require in-house synthesis—thus significantly - limiting their accessibility and clinical translation,^17^ where GMP-compliant materials would be required to meet regulatory standards for Human Advanced Therapy Medicinal Product (ATMP).^24^ At the present, linear polyethylenimine (l-PEI) is widely considered to be the “gold standard” among polycationic gene delivery agents due to its ease of use, cost-effectiveness and broad transfection efficiency.^25–28^ It is also available as a GMP-grade chemical (e.g., PEIpro-GMP commercialized by Polyplus). However, in the past PEI-based transfection of ARPE-19 cells typically resulted in low TE with significant cytotoxicity.^21,29,30^

Polymeric transfection starts with the formation of a polyelectrolyte complex (polyplex) between negatively charged genetic material (e.g., pDNA, mRNA) and positively charged polymers. Since these polyplexes are stabilized through electrostatic and hydrophobic interactions,^31^ their physicochemical properties—such as size, charge, and stability—directly influence TE and cell viability.^32^ Through adjustment of the molar ratio of polycation to genetic material (the so-called “N/P ratio”) a net-positively charged polyplex can be created which facilitates both interactions with the negatively charged cell membrane and eventual uptake by the cells.^33^ Factors such as the N/P ratio, complexing buffer, and incubation time are all important to account for in optimizing polyplexes formation.^26,34,35^ Indeed, optimizing the transfection protocol is critical for achieving high TE while preserving a high cell viability for a given cell line.^32,36,37^

Polyplexes preparation is typically done manually using techniques like pipetting and vortexing which, although simple, are regrettably prone to batch variations and operator inconsistencies which can lead to heterogeneous results across studies.^35,38–41^ Furthermore, even experienced operators often face challenges in achieving reproducible polyplex formation, causing significant variability in transfection efficiency. Yet current methods struggle to ensure consistent polyplex formation, particularly in large-scale applications.^35^ Reproducible, automated, and scalable polyplex production will be essential for future clinical applications^40^ and the field of microfluidics has already shown tremendous promise in improving the reproducibility of nanoparticles formation, including poly- and lipoplexes.^39–41^ Commercial T-junction systems, patented confined impinging jet mixer and pluggable platforms have been developed, but these still face limitations including high dead volume (up to 1 mL), suboptimal mixing rate, the need for high flow rates or limited flexibility and scalability (up and/or down).^38,40–45^ In addition, some microfluidic structures have been created by electro-micro milling or soft lithography methods (e.g., flow focusing generators, surface acoustic wave (SAW), herringbone or tesla micromixers, droplet generators, and/or hydrodynamic flow focusers) to produce poly- or lipoplexes microfluidically.^39,46–55^ Soft lithography has significant drawbacks, such as limited solvent compatibility, a fundamental reliance on cleanroom facilities, and the need for time-consuming manual assembly which also foreseeably opens up the door to errors.^40,46,56,57^ Soft-lithography-based microfluidic mixing structures (such as the T-mixers or a herringbone structures) also tend to exhibit comparatively poor mixing properties and low production rates (e.g., <0.167 mL/min) when compared against more advanced three-dimensional designs that integrated micromixer into the microfluidic system, like so-called HC mixers (named after a combination of H shaped and Chain mixer).^40,58,59^ These HC mixers ensure both quick (in a second) and thorough mixing of components (e.g., pDNA and polycation) even at low flow rates (50 μL/min) while also maintaining low shear stress (19.7 dyn/cm^2^ at 125 μL/min).^58^ However, producing such 3D structures using soft lithography requires assembling several layers, which once again increases manufacturing complexity.^58^ By contrast, high-resolution 3D printing offers an alternative that permits fast and flexible manufacturing of complex microfluidic devices without the need for cleanroom facilities.^56,60,61^ Recent studies have demonstrated the successful application of 3D-printed microfluidic systems for polyplex production, using methods such as stereolithography (SLA) and fused deposition modeling (FDM).^62–64^ However, these printing techniques remain relatively limited in structural complexity, while the absence of integrated screw-type connectors complicates handling and may lead to leakage issues.^40,62–65^ In most reported systems, connectors typically consist of glued-in cannulas or simply pushed-on tubes, which may be unreliable. To address these challenges, our group developed a 3D-printed microfluidic system with an integrated HC micromixer that is characterized by a low dead volume (<30 μL) and integrated 3D-printed connectors, thereby ensuring a direct and leakage-free operation.^58^ The connectors were printed directly onto the microfluidic chip, and are compatible with commercially available connectors. Using this system, we then successfully transfected Chinese hamster ovary cells in suspension (CHO_sus_) by directly mixing the cells with the pDNA and 25 kDa l-PEI (i.e., avoiding prior polyplex formation) in an approach that demonstrably outperformed the conventional manual technique.^66^

This study presents a multiparametric approach to optimizing TE and cell viability in ARPE-19 cells using commercially available l-PEI (25 kDa). The optimized protocol was transmuted into a semi-automated method for producing high-quality polyplexes by integrating a 3D-printed microfluidic system with a HC micromixer. The superior practicability of this system for robust polyplex production without an influence of an experimenter, in view of an efficient transfection of ARPE-19 cells, was assessed in comparison with polyplexes prepared using conventional manual techniques.

## Materials and Methods

Unless otherwise indicated, we used Greiner Bio-One (Frickenhausen, Germany)/Sarstedt (Nümbrecht, Germany) as the supplier for cell culture materials and Sigma-Aldrich (Taufkirchen, Germany) as the supplier for chemicals. The ARPE-19 cell line (immortalized retinal pigmented epithelial cells (RPE), CRL-2302) was obtained from ATCC (Manassas, USA). The following products were purchased from the following companies: Fetal calf serum (FCS) - Biochrom (Biochrom AG, Berlin, Germany) or VWR (Ismaning, Germany); Dulbecco’s Modified Eagle’s Medium (DMEM) - VWR (Ismaning, Germany); Dulbecco’s phosphate-buffered saline (DPBS) without Ca^2+^ and Mg^2+^, Trypsin/EDTA, and penicillin/streptomycin - Lonza (Visp, Switzerland) or VWR (Ismaning, Germany); Amphotericin B - Corning (NY, USA) or Biowest (Nuaillé, France); l-Glutamine - Gibco (Fisher Scientific, Schwerte, Germany) or stable l-Glutamine - Biowest (Nuaillé, France); staining dye peqGREEN - VWR (Ismaning, Germany); sterile ultrapure PCR water - Sigma-Aldrich (Taufkirchen, Germany) or VWR (Ismaning, Germany); 7-Aminoactinomycin (7-AAD) - Apollo Scientific (Bredbury, U.K.). The transfection medium Opti-MEM was purchased by ThermoFisher Scientific (Dreieich, Germany). Opti-MEM is based on Minimal Essential Medium (MEM) and contains proprietary amounts of insulin, transferrin, hypoxanthine, thymidine, GlutaMAX and trace elements.

HBG buffer (20 mM Hepes, 5 wt % glucose, pH 5.5) was prepared in-house and sterilized by filtration (Chromafil, CA-20/25­(S), 0.2 μm; VWR, Ismaning, Germany) or Filtropur S, 0.2 μm, Sarstedt (Germany). Linear PEI (l-PEI, 25 kDa) (Polysciences Europe GmbH, Eppenheim, Germany) stock solution was prepared in sterile ultrapure PCR water at 1.25 mg/mL.

For the microfluidic system: 2- and 20 mL syringes (Inject Luer Solo, B. Braun, Melsungen, Germany); syringe pump (AL-1000, World Precision Instruments, Sarasota, USA).

### Plasmid

pEGFP-N1 (4.7 kb) (Clontech Laboratories, Inc. (Mountain View, CA, USA)) encodes for an enhanced Green Fluorescent Protein (referred to as eGFP) and was amplified in *Escherichia coli* using standard laboratory techniques (LB medium supplemented with 30 μg mL^–1^ kanamycin). An EndoFree Plasmid Kit (Giga Prep/Maxi Prep) from QIAGEN (Hilden, Germany) was used for pDNA preparation (quality control: >80% supercoiled topology (agarose gel), and *A*_260_/*A*_280_ ≥ 1.8). Purified pDNA were solubilized in sterile ultrapure PCR water.

### Cell Line and Culture Conditions

The ARPE-19 cell line was cultured in DMEM supplemented with 10% (v/v) FCS, 4 mM (stable) l-Glutamine, 100 U/mL penicillin/streptomycin, and 2.5 μg/mL amphotericin B. This medium is referred to as D10. For cell maintenance, the cells were passaged two times a week with a starting cell density of 100,000 cells/mL and cultivated in a standard mammalian cell culture incubator at 37 °C, 5% CO_2_, 95% humidity. The cells were collected by trypsinization (5 min incubation time, 37 °C, 5% CO_2_, 95% humidity). For pre-equilibration, media were incubated for 1 h in the cell culture incubator.

### Determination of Cell Count and Viability

A LUNA-FL Dual Fluorescence Cell Counter (Logos Biosystems, Gyeonggi-do, South Korea) was used to determine cell count and viability of cells. Cells were stained with an Acridine Orange (AO, staining all cells)/Propidium Iodide (PI, staining dead cells) solution (Logos Biosystems, Gyeonggi-do, South Korea) according to the supplier’s instructions. Alternatively, cell number and viability were determined using a LUNA-II Automated Cell Counter (Logos Biosystems, Gyeonggi-do, South Korea). Cells were stained with Trypan blue (staining dead cells) solution (Logos Biosystems, Gyeonggi-do, South Korea) according to the supplier’s instructions.

### Design and Fabrication of the 3D-Printed Microfluidic Systems for Polyplex Formation

The microfluidic systems with the integrated HC micromixer were designed using SolidWorks (Dassault Systems Deutschland GmbH, Stuttgart, Germany) and then 3D-printed using a high-resolution MultiJet 3D printer (ProJet MJP 2500 Plus, 3D Systems, Rock Hill, SC, USA) with the following two materials: VisiJet M2S-HT90 (printing material) and VisiJet M2 Sup (support material). The postprocessing of the microfluidic systems was performed according to the methods previously described by Dehne et al.^66^ Briefly, after printing the support material was removed using a steam bath, followed by an ultrasonic oil bath, and then by flushing the channels with hot oil. Subsequently, any residual oil was eliminated through an ultrasonic water bath, after which the channels were also rinsed with hot water and detergent. Finally, the channels were rinsed sequentially with pure water, 80% ethanol, and then pure water yet again. The microfluidic system was finally autoclaved to ensure sterility for cell culture use.

The developed microfluidic systems for polyplex formation encompass two inlets: one for pDNA and l-PEI solutions (Chip_complexation_, dead volume: <30 μL, Figure 1A) or one for polyplex solution and Opti-MEM (Chip_dilution_, dead volume: 63.5 μL, Figure 1B). In both chip designs, the two incoming solutions merge into a single channel, where they are homogeneously mixed using an integrated HC micro mixer (Figure 1C).^58^ The resulting (diluted) polyplex solution is then pumped out through the outlet and collected in a reaction tube. To minimize any potential inaccuracies that could arise from using separate pumps for the incoming solutions (pDNA, l-PEI, polyplexes, and Opti-MEM), we utilized a single syringe pump to control their delivery into the chip. For a complete overview of the microfluidic setup, including the syringe pump and tubing, see Figure S1 in the SI.

**Figure 1:**
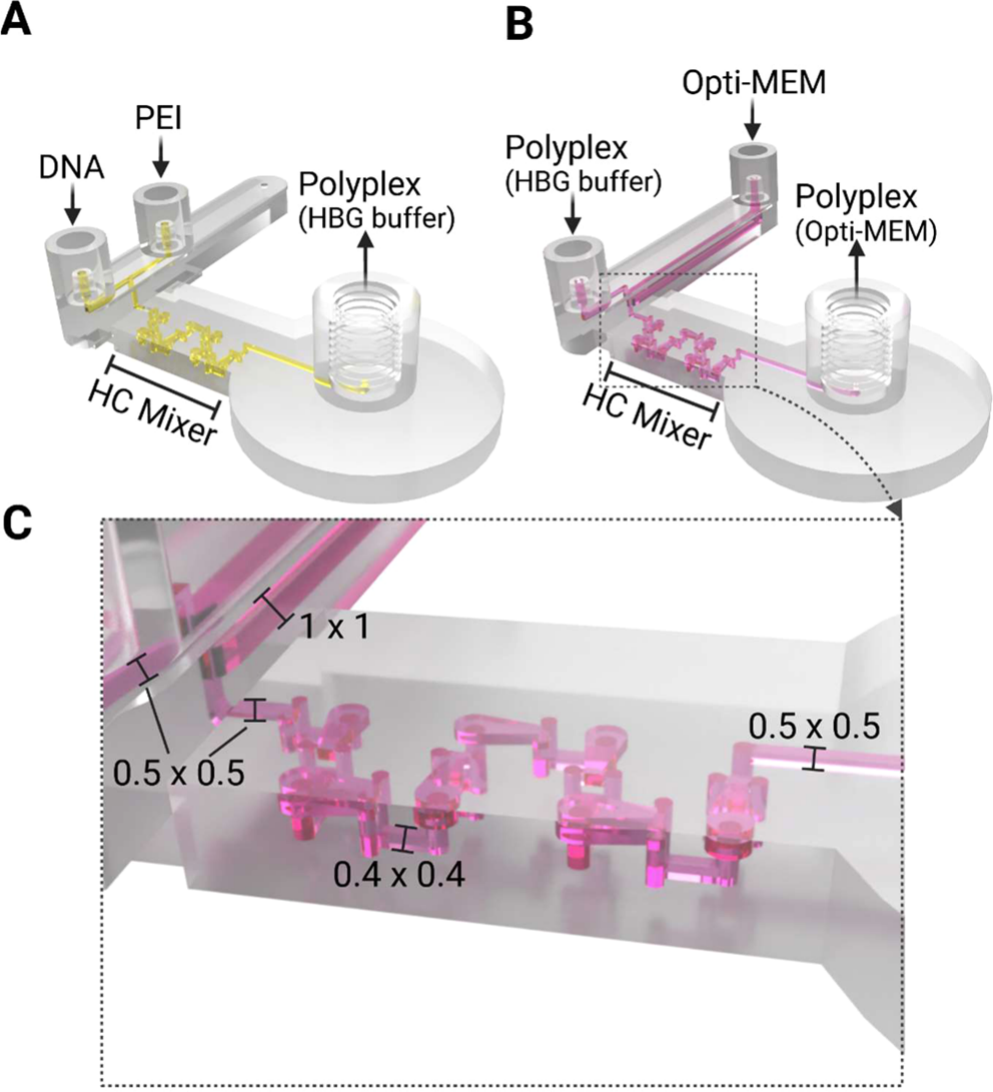
Illustration of the 3D-printed microfluidic systems (CAD design). A: Chip_complexation_, system for producing the polyplexes in HBG buffer. B: Chip_dilution_, system for mixing the preformed polyplexes with Opti-MEM during the dilution step. C: Close-up of the HC micromixer with dimensions (all in mm). For full dimensions of the HC micromixer see Enders et al. 2019.^58^

### Transfection

One day prior to transfection, ARPE-19 cells were harvested by trypsinization following standard laboratory protocols, including trypsin inactivation by growth medium. The cells were then seeded at 2 × 10^5^ and 8 × 10^4^ cells per well in 6- and 12-well plates, respectively, and incubated for 24 h in the cell culture incubator (37 °C, 95% humidity, 5% CO_2_). On the day of transfection, polyplexes were prepared either by maintaining a constant amount of pDNA while adjusting the l-PEI concentration, or otherwise by maintaining a constant amount of l-PEI while adjusting the pDNA concentration to establish the desired N/P ratios. N/P-ratios were calculated according to eq 1.

1 $$\frac{N}{P}=\frac{\left(\mu L\;l‐\mathrm{PEI}\;\mathrm{stock}\;\mathrm{solution}\times N\right)}{\left(\mu g\;\mathrm{pDNA}\times p\right)}$$

with *N* = concentration (mM) of nitrogen residues in l-PEI and *p* = nmoles phosphate in pDNA. Note: 1 μg of pDNA contains 3 nmoles of anionic phosphate.

Figure 2 illustrates the different methods of preparing the polyplexes: manual, microfluidic, and semi-automated microfluidic approaches. Regardless of the method employed, the polyplex preparation always involves two sequential steps. In the first step, pDNA and polymer are mixed in salt-free HBG buffer to form polyelectrolyte complexes (i.e., polyplexes) in what is referred to as the “Complexation step.” Then in the second step, the polyplexes are diluted in a salt-containing and serum-free transfection medium (Opti-MEM), which is referred to as the “Dilution step”.

**Figure 2:**
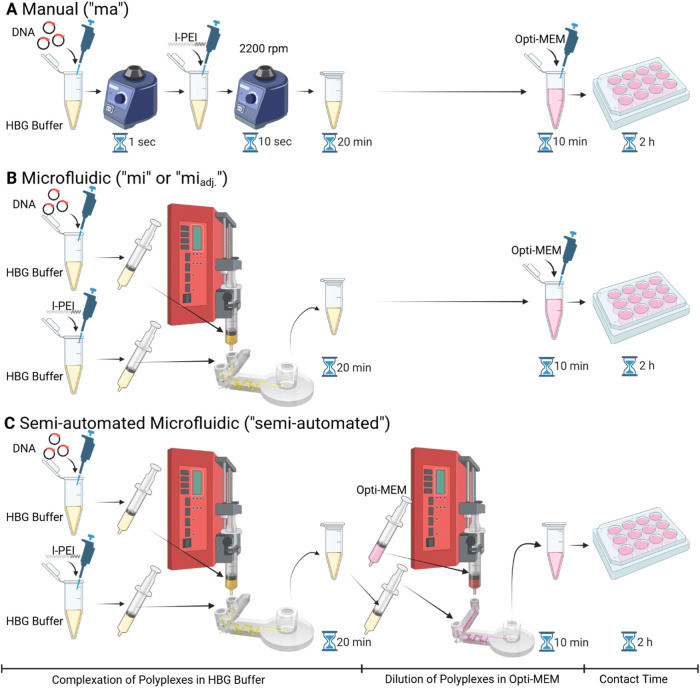
Overview of different methods for polyplex formation prior to cell transfection: manual (A) and microfluidic-assisted approaches (B, C). (A) Polyplexes are produced manually by pipetting and vortexing (PP_ma_). (B) Polyplexes are produced using the Chip_complexation_ microfluidic system (Setup B “mi”; pDNA: 99 μg/mL, l-PEI: 128 μg/mL, both in 2 mL syringes, PP_mi_) or with adjusted concentrations (Setup B “mi_adj_.”; pDNA: 52.1 μg/mL, l-PEI: 67.4 μg/mL, both in 2 mL syringes, PP_mi adj._). In both cases, the subsequent dilution step with Opti-MEM is performed manually. (C) Semi-automated microfluidic transfection method. Both polyplex formation (Setup C “semi-automated”; pDNA: 52.1 μg/mL, l-PEI: 67.4 μg/mL, both in 2 mL syringes, Chip_complexation_) and dilution step with Opti-MEM (2 mL for polyplexes mixture, 20 mL syringe for Opti-MEM, Chip_dilution_) are performed in microfluidic systems (PP_semi-automated_). (created with Biorender.com).

#### Conventional Manual Polyplexes Generation

Figure 2A: For manual transfection (Setup A “ma”), polyplexes were prepared by pipetting and vortexing, as previously established in our group.^37,67^ Briefly, this process involves mixing the appropriate amount of pDNA in a final volume of 50 μL (for transfection in 12-well plates) or 200 μL (for transfection in 6-well plates) of HBG buffer, with the required amount of polycation to achieve the desired N/P ratio. The polycation solution is then added in a single drop and the mixture was vortexed for 10 s. Following a 20 min incubation period, unless otherwise stated the polyplex mixture is diluted with 450 μL (for 12-well plate) or 1 mL (for 6-well plate) of Opti-MEM. These polyplexes are referred to as PP_ma_.

#### Microfluidic-Assisted Polyplexes Generation

Figure 2B,C: In a first approach (Setup B “mi”), the complexation step was automated, while the dilution step was performed manually. For complexation pDNA (99 μg/mL) and l-PEI (128 μg/mL), both were prepared in HBG buffer and then they were loaded in two separate 2 mL syringes, which were secured in an in-house 3D-printed syringe holder attached to a single syringe pump (Figure S1A). The two solutions were pumped at defined flow rates (1−6 mL/min) through the microfluidic system (Chip_complexation_ (Figure 1A)). The resulting mixture (50 μL for 12-well plates, if not otherwise stated) was incubated at room temperature for 20 min in a microcentrifuge tube, after which time the polyplexes were manually mixed with Opti-MEM in a 10-fold dilution step (e.g., for 500 μL of transfection mixture: 50 μL polyplexes + 450 μL Opti-MEM) via pipetting (Figure 2B). These polyplexes are referred to as PP_mi_.

To assess whether concentration adjustments made in the Setup C “semi-automated” (where both complexation and dilution were automated, see below) affected polyplex formation and transfection efficiency, a variation of Setup B “mi” (referred to as Setup B “mi_adj._”) was tested. Here, pDNA and l-PEI solutions were prediluted by about 2-fold before being loaded into the 2 mL syringes. Polyplexes were then formed using the same procedure as above. These modified polyplexes are referred to as PP_mi adj._.

In the second approach (Setup C “semi-automated”), both the complexation and dilution steps were automated. To enable microfluidic dilution while preserving the final concentrations for the cells, the DNA and PEI concentrations in the complexation step were adjusted based on the feasible syringe diameter ratios for the polyplex solution and Opti-MEM, which determine the dilution rates in the dilution step. Specifically, the pDNA concentration was set to 52.1 μg/mL, and the l-PEI concentration to 67.4 μg/mL (i.e., as for Setup B “mi_adj._” above). The polyplex complexation was the same as in described for Setup B “mi_adj._” with microfluidic Chip_complexation_ (Figure 1A and Figure S1A). After polyplex formation, the mixture was transferred manually into a new 2 mL syringe, while a 20 mL syringe was filled with Opti-MEM. Both of these syringes were then placed in the 3D-printed holder (Figure S1B), and the solutions were pumped through the Chip_dilution_ microfluidic system (Figure 1B). The final dilution ratio of Opti-MEM to polyplexes was 4.3:1 (i.e., for 500 μL of transfection mixture: 95.2 μL polyplexes + 404.8 μL Opti-MEM). These polyplexes are referred to as PP_semi-automated_.

As a negative control, the cells underwent a respective mock transfection (referred to as “Mock”) where they were incubated solely with the complexation buffer (HBG) and the dilution medium (HBG buffer + Opti-MEM), without pDNA. For microfluidic polyplex formation (PP_mi_ and PP_mi adj._) HBG and for polyplex dilution PP_semi-automated_ HBG and Opti-MEM were also prepumped through the system beforehand.

Regardless of the polyplexes complexation and dilution methods, the polyplexes were incubated for 10 min at room temperature after dilution in Opti-MEM. During this time, the cells were washed twice with DPBS. The diluted polyplex mixture was then added dropwise to the cells and gently distributed by rocking the plates before placing them back into the cell culture incubator. The total transfection volume was 1 mL for 12-well plates and 2 mL for 6-well plates or 0.5 mL for 12-well plates for transfections performed at reduced volumes. After an incubation period of up to 4 h (contact time), the supernatant was removed and replaced with either 1 mL (12-well plate) or 2 mL (6-well plate) of D10 medium. The cells were returned to the cell culture incubator for up to 48 h (recovery time) before being analyzed by flow cytometry. For flow cytometry analysis, cells were harvested by trypsinization, resuspended in DPBS and counterstained with propidium iodide (PI, 1 μg/mL) or 7-AAD (1 μg/mL) to identify dead cells. The gating strategy used for analysis of the transfection efficiency (% of enhanced Green Fluorescent Protein (eGFP) -positive cells) is shown in Figure S2 in the Supporting Information.

### Analytics

#### Gel Retardation Assay

A gel retardation assay was carried out to determine the N/P-ratio required for complete DNA complexation (i.e., net charge compensation). Polyplexes were prepared in 50 μL HBG using 2 μg of pDNA and varying amounts of l-PEI to reach the indicated N/P ratio. The mixtures were vortexed for 10 s and then incubated at room temperature for complexation. After 20 min of incubation, 5 μL 10× loading buffer (60% glycerol, 10 mM Tris-HCl pH 7.6, 60 mM EDTA, 0.03% bromophenol blue) was added to each sample. Subsequently, 15 μL of the mixtures were loaded onto a 1% (w/v) agarose gel, and electrophoresis was conducted in Tris-acetate-EDTA pH 8.1 buffer at 90 V for 90 min. The gels were stained with peqGREEN (60 ng/mL), and pDNA was visualized under ultraviolet (UV) light (254 nm).

#### Particle Size and Zeta Potential Measurements

To assess the particle size and charge, dynamic light scattering (DLS) and zeta potential measurements were performed using a Litesizer 500 (Anton Paar, Ostfildern-Scharnhausen, Germany) and the reusable cuvette Univette Low Volume. Polyplexes were prepared as described above in the Transfection section. The N/P ratio was adjusted by varying the pDNA amount, while maintaining a constant polymer concentration. Hydrodynamic radii were followed for 10 min in HBG. All incubations and measurements were performed at room temperature. For analysis, we focused on peak 1 intensity, representing the hydrodynamic diameter of the smallest detected peak in case of polydisperse distributions. The particle charge was calculated using the Helmholtz-Smoluchowski equation.^68^ All used parameters for DLS measurements (hydrodynamic diameter and zeta potential) are provided in the Supporting Information (Table S1).

#### Determination of the Transfection Efficiency and Viability

The transfection efficiency (TE) was assessed by measuring eGFP fluorescence using flow cytometry (Cytomics FC500, dual laser (488 nm, 635 nm) or Cytoflex S (488 nm, 638 nm), both from Beckman Coulter, Krefeld, Germany. Forward scatter (FSC), side scatter (SSC), green fluorescence (GFP, em. 525 nm), and red fluorescence (PI, em. 620 nm and 7-AAD, em. 690 nm) were recorded. Negative controls (i.e., mock-transfected cells) were used to set the measurement parameters. Data were collected from at least 10,000 events. Cells were initially evaluated by scatter properties (FSC/SSC, linear scale) to select the ARPE-19 population (Gate: “ARPE-19”) and to exclude aggregates and apoptotic cells. The gating strategy for analyzing the transfected ARPE-19 cells is shown in Figure S2. The relative eGFP fluorescence of the gated cells was measured, allowing for a statistical quantification of TE in the “ARPE-19” population. eGFP-expressing cells were defined as those with fluorescence greater than the autofluorescence of the mock-transfected cells. Simultaneously, red fluorescence intensity (PI/7-AAD) was used to assess cell viability. Histograms and dot plots of the respective fluorescence intensities (log scale) were used to classify eGFP levels of expression: Low producers: fluorescence intensity between 10^3^–10^4^ (or 2 × 10^4^–2 × 10^5^); Middle producers: fluorescence intensity between 10^4^–10^5^ (or 2 × 10^5^–2 × 10^6^); High producers: fluorescence intensity exceeding 10^5^ (>2 × 10^6^). The values in parentheses correspond to measurements acquired from a different flow cytometry instrument with distinct sensitivity parameters. Representative dot plots and histograms are showed in Figure S2. The gates were set using the same criteria for both flow cytometers. Flow cytometry data were evaluated using FlowJo software v 10.8.1 (Tree Star, Stanford University, Stanford, CA, USA, 2016) or with the CytExpert software version 2.6 (Beckman Coulter, Krefeld, Germany).

#### Statistical Analysis

Group data are presented as mean ± standard deviation (SD). Unless otherwise stated, *n* indicates the number of independent experiments. Statistical analyses were conducted using one-way and two-way ANOVA with Bonferroni correction, performed using the OriginPro software (version 2024, OriginLab, Northampton, MA, USA). The static significance was determined as follows: * (*p* < 0.05); ** (*p* < 0.01); *** (*p* < 0.001).

## Results and Discussion

### Adapting Standard Nonviral Polyfection to the Needs of ARPE-19 Cells

We initially evaluated the standard method recommended by the l-PEI manufacturer and outlined in the pertinent literature, e.g., Raup et al. 2016 ^69^ or Kumar et al. 2022 ^70^ for 25 kDa l-PEI, widely regarded as the “gold standard” for polycationic transfection, in transfecting ARPE-19 cells. The first step recommended for optimizing transfection efficiency (TE) is adjusting the N/P ratio. Following this standard procedure, polyplexes were prepared using a constant amount of pDNA (3 μg per well) and varying amounts of l-PEI to achieve the desired N/P ratio. The cells were transfected in 6-well plates, with an initial cell density of 2 × 10^5^ cells/well on the day of seeding, and a contact time of 4 h between polyplexes and cells. Results for transfection efficiency (TE) and cellular viability after 24 h of recovery time are shown in Figure 3.

**Figure 3:**
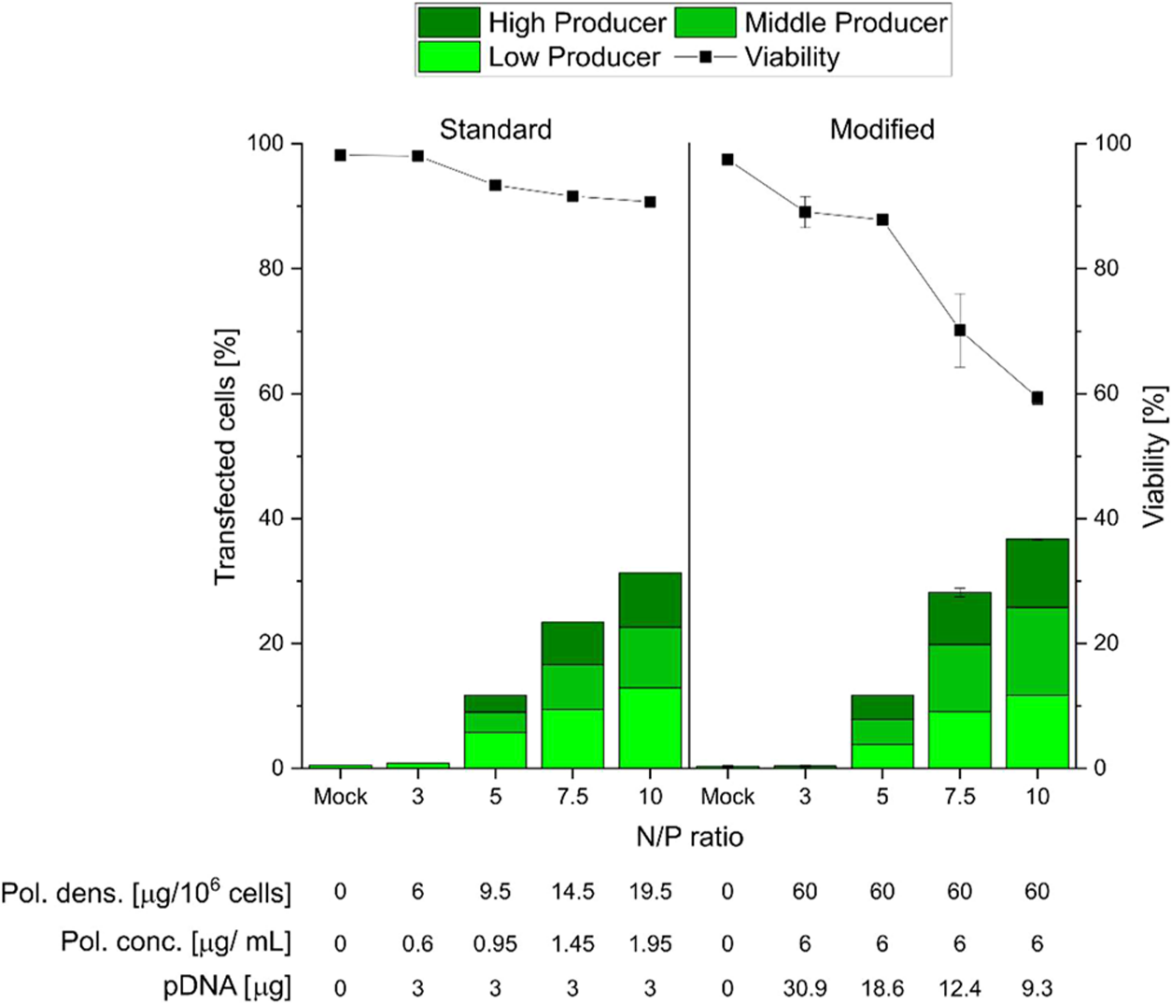
Influence of the N/P ratio on TE and viability at a constant (“Standard”) and varying (“Modified”) pDNA amount per well. Total cells: 2 × 10^5^, 6-well plates, transfection volume: 2 mL (0.2 mL polyplex solution), contact time: 4 h, recovery time post-transfection: 24 h. Lines are guides to the eye. For “Standard”, *n* = 1; for “Modified”, data represent mean values ± SD with *n* ≥ 2. Pol. conc.: polymer concentration; Pol. Dens.: polymer density. eGFP expression levels were classified based on fluorescence intensity (log scale) using histogram representations of the flow cytometry data. Detailed criteria are provided in the Materials and Methods section and Figure S2.

The relatively low TE values (≤30%) are in line with previously reported results for l-PEI and jetPEI in ARPE-19 cells (TE < 22%),^18,29,70^ and they are too low for most applications. At N/P ratios ≤5, most transfected cells are low producers, but as the N/P ratio increases, the fraction of middle and high producers increases, with N/P ratios of 7.5 and 10 yielding comparable results. The high cell viability (>90%) is also in line with the common experience that high TE is usually accompanied by low viabilities. Moreover, these results indicate that polymer concentrations over the entire investigated range (i.e., up to 2 μg/mL) are well-tolerated by the cells. Notably, such concentrations are more than 5-fold below the LD_50_ value (10.1 μg/mL) determined by us for free 25 kDa l-PEI in ARPE-19 cells using the MTT assay (Figure S3).

Although even the highest l-PEI concentrations were below the LD_50_, previous studies in our group have shown that the changing polycation concentration and in consequence the polymer density (expressed as μg of polymer per 10^6^ cells)—that is experienced by the cells when the N/P ratio is adjusted via the polycation concentration can adversely impact the transfection outcomes, including both TE and cell viability.^36,37^ More consistent results have previously been obtained when adjusting the N/P ratio by varying the amount of pDNA while keeping the amount of l-PEI constant. This has been shown to improve TE while simultaneously preserving high cell viability, even in cells that are resistant to transfection.^37,67,71^ The corresponding results in case of the ARPE-19 cells are summarized in Figure 3, “Modified”. In addition, in this set of experiments we increased the polymer concentration to 6 μg/mL (i.e., still well below the LD_50_) based on prior evidence suggesting that increasing polymer concentration can enhance TE, as shown for PDMAEMA-based transfection reagents.^36,37^ This corresponds to 60 μg of polymer per 10^6^ cells at all investigated N/P ratios.

The “Modified” approach yielded higher TE compared to the “Standard” approach, reaching up to 40% transfected cells at N/P 10 (Figure 3). Additionally, this method resulted in a more favorable distribution of transgene expression, with a slight increase of middle and high producers observed. However, cell viability was somewhat reduced at higher N/P ratios, even though it remained ≥ 60% across all tested conditions. It is unlikely that this effect is related to the overall polymer concentration or polymer density, since both of those parameters were kept constant in the experiments. However, at higher N/P ratios (which correspond to lower DNA amounts in this case), the level of excess free polymer is higher. Previous studies have reported that noncomplexed PEI is more toxic to cells than it is when it exists in complexed form.^72–74^ Moreover, since the observed decrease in cell viability correlates with an increase in TE and a higher proportion of high producers, the eGFP expression itself may exert a toxic effect on the ARPE-19 cells; intracellular toxicity of eGFP has, after all, been observed and documented in earlier studies.^75^ Overall, the “Modified” approach presents a more effective strategy for transfecting ARPE-19 cells with l-PEI, although the impact of polymer density and polymer concentration on cell viability obviously must be managed in order to ensure optimal performance in particular for *in vivo* applications.

### Influence of Contact Time and Reaction Volume on Transfection of ARPE 19 Cells

It has been shown that shortening the contact time between cells and the transfection mixture improved cell viability while keeping TE at sufficient levels.^67,76,77^ Additionally, reducing the reaction volume - and thereby increasing the local polyplex concentration – also has the potential to significantly improve TE in suspension cells.^37,78^ For adherent cells, a reduction in transfection volume may decrease the diffusion distance for polyplexes, thereby shortening the time required for them to reach the cells. In consequence, ARPE-19 cells were transfected using a polymer density of 80 μg per 10^6^ cells and an N/P ratio of 5. Transfection volumes of 0.5 and 1 mL were tested, with contact times of 2 and 4 h. Increasing the polymer density to 80 μg per 10^6^ cells was expected to further increase the detectability of subtle trends in transfection efficiency (TE), level of gene expression, and cell viability. Lowering the N/P ratio from 10 to 5 was a strategic modification designed to reduce cytotoxicity. These changes allowed us to evaluate the effects of shorter contact times and reduced transfection volumes under conditions where the chance of observing measurable outcomes was optimized. Additionally, the switch to a 12-well plate while using lower cell density (8 × 10^4^ cells/well) ensured compatibility of the cultivation vessels with the reduced transfection volumes, preserving experimental consistency. The results are summarized in Table 1.

**Table 1:** Analysis of the Influence of Contact Time and Transfection Volume on TE and Cell Viability

		TE [%]		cell viability [%]	
	contact time	0.5 mL^a^	1 mL^b^	0.5 mL	1 mL
l-PEI	2 h	31.8 ± 1.4	26.0 ± 0.6	73.2 ± 0.9	83.5 ± 0.5
	4 h	24.6 ± 6.9	23.9 ± 1.2	77.4 ± 12.6	89.2 ± 0.2

a Polymer concentration: 12.8 μg/mL.

b Polymer concentration: 6.4 μg/mL.

Independently of the transfection parameters, TE never exceeded 32% while cell viability consistently remained above >73%. Neither the contact time nor the transfection volume was found to have a statistically significant impact on TE and cell viability. However, a nonstatistically significant trend suggested that reducing both volume and contact time might enhance TE. Reducing transfection volume could potentially enhance polyplex sedimentation by decreasing the vertical diffusion distance, thereby leading to faster accumulation at the cell surface. Prior research has demonstrated that gravitational settling plays a role in determining TE by influencing polyplex accumulation at the cell surface.^32^ Reducing the transfection volume, which effectively doubled the polymer concentration - also resulted in a nonstatistically significant trend toward decreased cell viability. We attribute this decrease to the polymer concentration exceeding the LD_50_ range for l-PEI. Despite this, cell viability still consistently exceeded 70%. Overall, compared to the results in Figure 3 at N/P 5 (TE 12%, viability 88%), performing the transfection in 12-well plates with 8 × 10^4^ cells on the day of seeding using a total transfection volume of 0.5 mL and a 2-h contact time between cells and polyplex improved TE 2.7-fold to 31.8% with only a slight reduction in viability. As a result, this setup was used in all subsequent experiments.

### Influence of Polymer Density, N/P Ratio, and Recovery Time on Transfection Outcomes

The results presented above (compared Figure 3 and Table 1) suggest that increasing the polymer density has a positive effect on TE without excessively affecting cell viability. To test this hypothesis, we evaluated the effect of polymer densities in a range of 20 to 100 μg polymer per 10^6^ cells (12-well plate format). Additionally, given that ARPE-19 cells have a doubling time of approximately 55 h,^79^ we assessed the impact of increasing the recovery time post-transfection by analyzing TE and cell viability at 24 and 48 h. The corresponding results are summarized in Figure 4.

**Figure 4:**
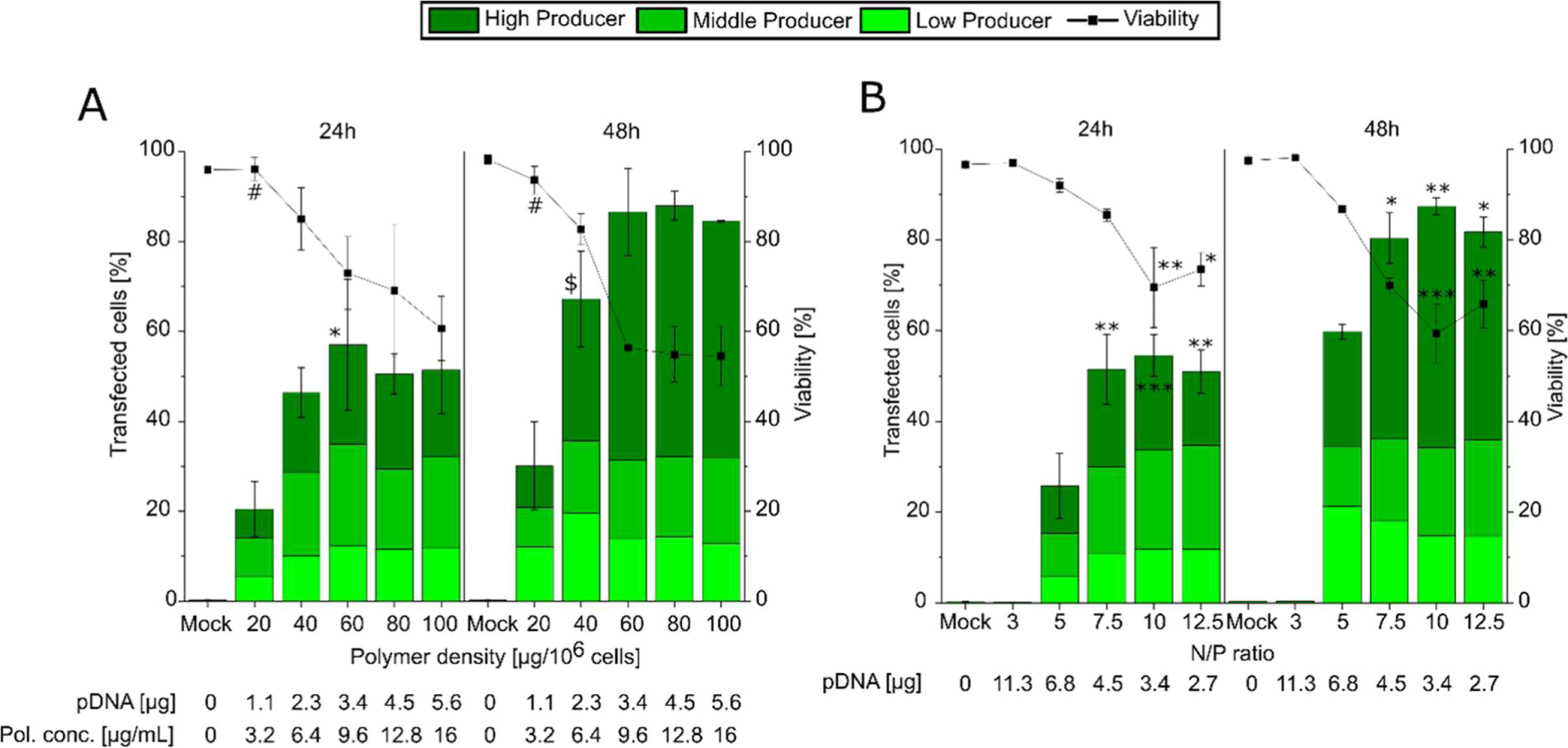
Evaluation of polymer density (A) and N/P ratio (B) on transfection outcomes. (A) N/P ratio constant at 10, polymer density variable. (B) N/P ratio varied, polymer density (60 μg polymer per 10^6^ cells) and concentration (9.6 μg/mL) constant. Total cells: 8 × 10^4^ per well, 12-well plate, transfection volume 0.5 mL (0.05 mL polyplex solution), contact time 2 h. Recovery time: Analysis was conducted after 24 and 48 h of recovery post-transfection. Lines serve as guides to the eye. Data represent mean values ± SD *n* ≥ 2. (A) Statistically significant differences in cell viability to 100 μg polymer per 10^6^ cells are indicated as # (*p* < 0.01), and TE to 20 μg polymer per 10^6^ cells is indicated as * (*p* < 0.05), $ (*p* < 0.01). In panel (B) statistical significance of cell viability and TE compared to N/P 5 is indicated as * (*p* < 0.05) ** (*p* < 0.01) *** (*p* < 0.001).

A gradual increase in TE was observed with increasing polymer density, reaching up to 60%. However, TE plateaued at approximately 40 μg of polymer per 10^6^ cells. Beyond this threshold, TE values remained stable with no statistically significant differences observed between 40 and 100 μg of polymer per 10^6^ cells. Cell viability, however, showed a dose-dependent decrease, reaching 55% at a polymer density of 100 μg polymer per 10^6^ cells. Extending the recovery time post-transfection to 48 h enhanced TE approximately 1.5-fold, achieving levels as high as 80% while cell viability remained above 58%. At polymer densities of 40 μg/10^6^ cells or higher, the proportion of high-producer cells increased significantly—by 3-fold—especially when the recovery time was extended to 48 h, indicating efficient delivery and robust transgene expression (Figure 4A). No significant difference in viability was observed between 60 μg and 100 μg polymer/10^6^ cells after 24 and 48 h recovery time, even though these densities correspond to polymer concentration ≥ LD_50_ value for l-PEI. This was rather unexpected, since previous studies conducted on other cell lines had suggested that increased polymer density and concentration typically contribute to higher cell mortality.^37,67^ ARPE-19 cells (with an average size of approximately 15 μm, as measured using the Luna II automated counter) may exhibit a surface saturation effect at polyplex/polymer density above 60 μg polymer/10^6^ cells. A polymer density of 60 μg polymer/10^6^ cells corresponds to approximately 1.4 × 10^9^ polymer chains per cell, detailed calculation is provided in the Supporting Information. We hypothesize that beyond this threshold, additional polyplexes/free polymers could fail to interact with the cells, resulting in no further impact on cellular viability.

In the next optimization step, we investigated whether cell mortality could be reduced by varying the N/P ratio at polymer density corresponding to a polymer concentration close to the LD_50_. Transfections were conducted at N/P ratios ranging from 3 to 12.5 at a polymer density of 60 μg polymer/10^6^ cells (equivalent to a polymer concentration of 9.6 μg/mL); this density was chosen as it represented the lowest polymer density that achieved the highest TE according to Figure 4A. Transfection outcomes were evaluated after 24 and 48 h of recovery and are presented in Figure 4B. Consistent with the previous results (Figure 3), the N/P ratio was found to significantly impact transfection outcomes. At an N/P ratio of 3, cell viability was similar to that of mock-transfected cells, with no evidence of transfection observed at any recovery time. This is likely due to incomplete compensation of the pDNA charge at this ratio, as confirmed by gel retardation assay (Figure S4). TE was observed to improve with increasing N/P ratios, plateauing at an N/P ratio 7.5, where a higher proportion of high-producer cells was observed with cell viability remaining ≥60%. Extending the recovery time further enhanced TE. Specifically, at N/P 5 the TE increased by 2.3-fold, while higher N/P ratios showed a 1.6-fold improvement. TE and viability were comparable to those observed when varying polymer densities at a fixed N/P ratio of 10 (Figure 4A). One noticeable difference was that the cell viability consistently exceeded 70% after 24 h of recovery and only slightly decreased when the recovery time was extended to 48 h although this reduction was not statistically significant. Overall, these findings indicate that varying the N/P ratio for a preselected polymer density led to only marginal changes in TE and cell viability, provided that the pDNA charge was fully compensated.

The time-dependent effects observed in both panels indicate that TE improves over time, while cell viability remains unchanged. Consistent with earlier hypotheses, higher eGFP expression levels correlated strongly with increased cytotoxicity (Figure S5). The best TE outcomes were achieved with an N/P ratio of 7.5 or 10, using a polymer density of 60 or 40 μg polycation per 10^6^ cells. A maximum TE of ca. 70 to 80% was attained, along with ca. 70% cell viability, outperforming by nearly 4-fold the best previously published results. The observed increase in high producers under optimal conditions (i.e., N/P ratio, polymer density, polymer concentration) suggests that volume reduction during transfection and extended recovery time not only improve delivery efficiency but also enhance transgene expression levels. Of note, even though the reduced transfection volume is still somewhat high, it still falls within the range of volumes typically injected subretinally (300 μL) in clinical trials.^80^ Given that a polymer density of 60 μg polycation per 10^6^ cells—approaching the LD_50_ of l-PEI—did not result in a significant improvement in transfection outcomes, a lower polymer density of 40 μg polycation per 10^6^ cells (corresponding to a concentration below the LD_50_ of l-PEI) was selected for subsequent experiments.

### Physicochemical Properties of Manual and Microfluidic Produced Polyplexes

Polyplex preparation protocols proposed in this contribution are based on a two-step method developed in the past by our group.^36^ Standardization of polyplex preparation was attempted based on a custom-built 3D-printed microfluidic system previously developed in our group.^66^ The automated operation of this system is expected to minimize batch-to-batch variations and eliminate operator-induced inconsistencies associated with manual handling.

In setting up the microfluidic system, first the most suitable flow rate for the automated production of polyplexes was identified. In this context, the flow rate of pumping pDNA and l-PEI solutions into the HC micromixer was incrementally increased from 1 to 6 mL/min. Polyplexes were prepared at an N/P ratio of 10, a charge ratio that was chosen for its ability to achieve full charge compensation of the negatively charged pDNA - as demonstrated by the gel retardation assay (Figure S4). The N/P ratio was adjusted by varying the pDNA amount at a fixed polymer concentration (64 μg/mL), following the protocol developed previously in our group.^36^ pDNA and l-PEI solutions (Setup B “mi”) were pumped at the indicated flow rate into the HC micromixer (Chip_complexation_, Figure 1A). The residence time of the pDNA-l-PEI mixture in the microfluidic system ranged from 0.3 to 1.8 s, depending on the flow rate. Subsequently, the mixture was pumped directly into microcentrifuge tubes and then incubated for 20 min for polyplexes formation. To assess the impact of microfluidic preparation on the physicochemical properties of polyplexes, polyplexes are also prepared manually as described in the method section part “Transfection - Conventional manual polyplexes generation *”*. Specifically, 7.0 μg of l-PEI was added to 5.4 μg of pDNA in HBG buffer, and the mixture was vortexed for 10 s (total volume 110 μL). Polyplexes were analyzed by dynamic light scattering (DLS) and zeta potential measurements at two stages: (1) after initial complexation in HBG buffer, i.e., at the moment of leaving the HC micromixer, and (2) during the first 10 min of incubation in microcentrifuge tubes. Because no significant differences within the treatment group were observed during the first 10 min, and the differences between samples gradually diminished over time, only the first 10 min were analyzed. The results are depicted in Figure 5. In addition, the results of the polydispersity index can be found in Figure S6.

**Figure 5:**
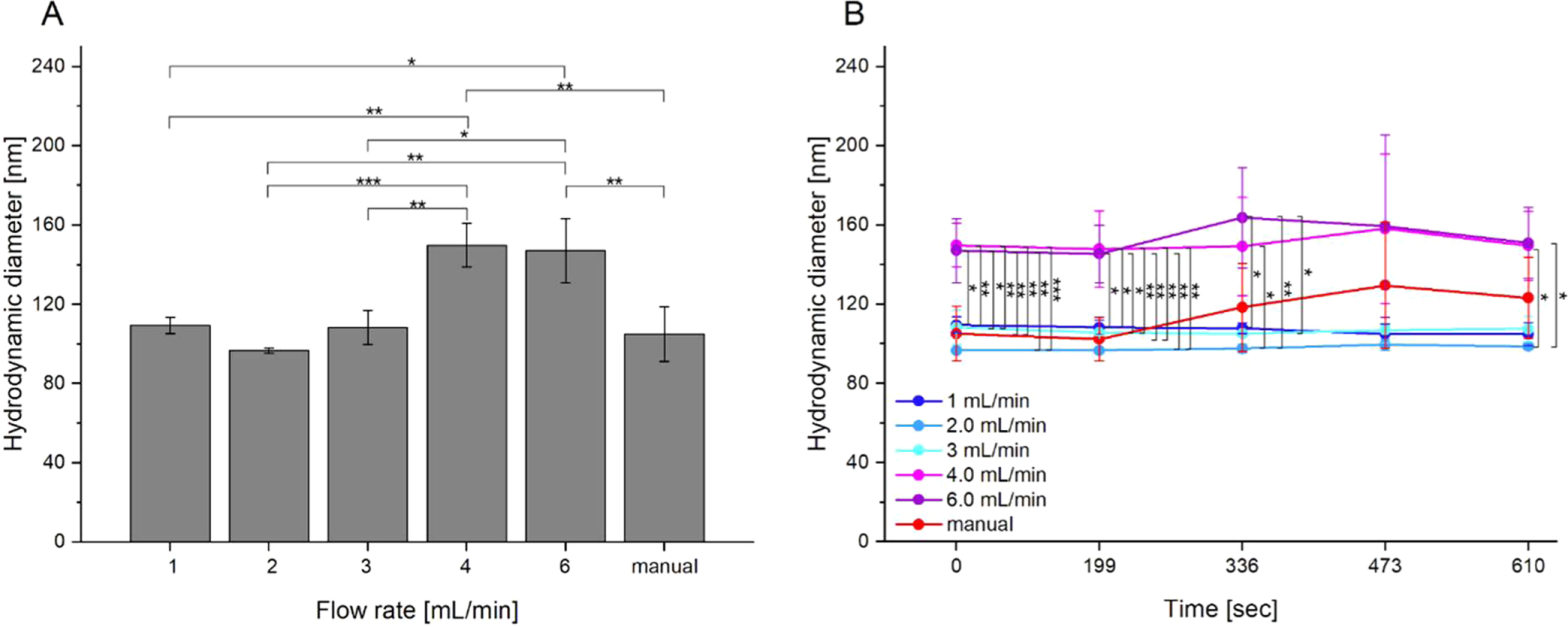
Hydrodynamic diameter of the polyplexes in HBG buffer at the moment of leaving the Chip_complexation_ (A) and during the initial complexation step in microcentrifuge tubes (B). Polyplexes were prepared at an N/P ratio of 10 via standard manual procedure (“manual”) and via microfluidic system (Setup B “mi”) at different flow rates. pDNA amount: 5.44 μg, l-PEI: 7.04 μg. Total volume: 110 μL. Data represents mean values ± SD with *n* = 3. *p ≤ 0.05; **p ≤ 0.01; ***p ≤ 0.001.

Microfluidically produced polyplexes at flow rates between 1 and 3 mL/min displayed a comparable average size of 104.7 ± 6.7 nm, which evidenced little change over time during incubation. Increasing the flow rate to 4 and 6 mL/min resulted in a statistically significant 1.4-fold increase in size, reaching an average of 148.5 ± 2.1 nm (Figure 5A). By comparison, the manually prepared polyplexes had an initial average size of 105 ± 13.8 nm (i.e., in the same range as those produced at flow rates of 1 to 3 mL/min). In contrast to the microfluidic ones, the size of the manually generated polyplexes did change with time and increased to 123 nm during the 10 min incubation (Figure 5B), illustrating the impact of secondary aggregation dynamics in that preparation. Importantly in view of our goal of reproducible polyplex production, significantly lower standard deviations were observed for polyplexes prepared at the lower flow rates.

Next, the impact of the N/P ratio on the physicochemical properties of polyplexes was examined in both salt-free HBG buffer and after dilution in salt-containing Opti-MEM (NaCl concentration according to the supplier: 116 mM). This setup was based on the fact that polyplexes (which are formed by electrostatic interactions between pDNA and l-PEI) are influenced by salt concentration, making it crucial to study their behavior in both environments. A flow rate of 2 mL/min was selected for microfluidic polyplex preparation (dubbed PP_mi_), as the polyplexes produced with an N/P ratio of 10 at that flow rate had been similar in size – at least initially – to the manually prepared ones (dubbed PP_ma_).

The N/P ratio was varied by adjusting the pDNA amount while maintaining a fixed polymer concentration (64 μg/mL). N/P ratio greater than 5 was chosen because, at N/P 5, transfection efficiency remains low, while ratios up to 20 were selected to find out how less pDNA is needed for high transfection efficiencies and to ensure larger differences between the samples in order to facilitate observation of any differences in physiochemical properties. Initially, the size and charge of the polyplexes were measured after a 20 min complexing step in HBG buffer; these parameters were then reassessed following the dilution step in Opti-MEM, after a 10 min incubation (i.e., polyplexes, which are then used for transfection). The results are shown in Figure 6. In addition, the results of the polydispersity index can be found in Figure S7.

**Figure 6:**
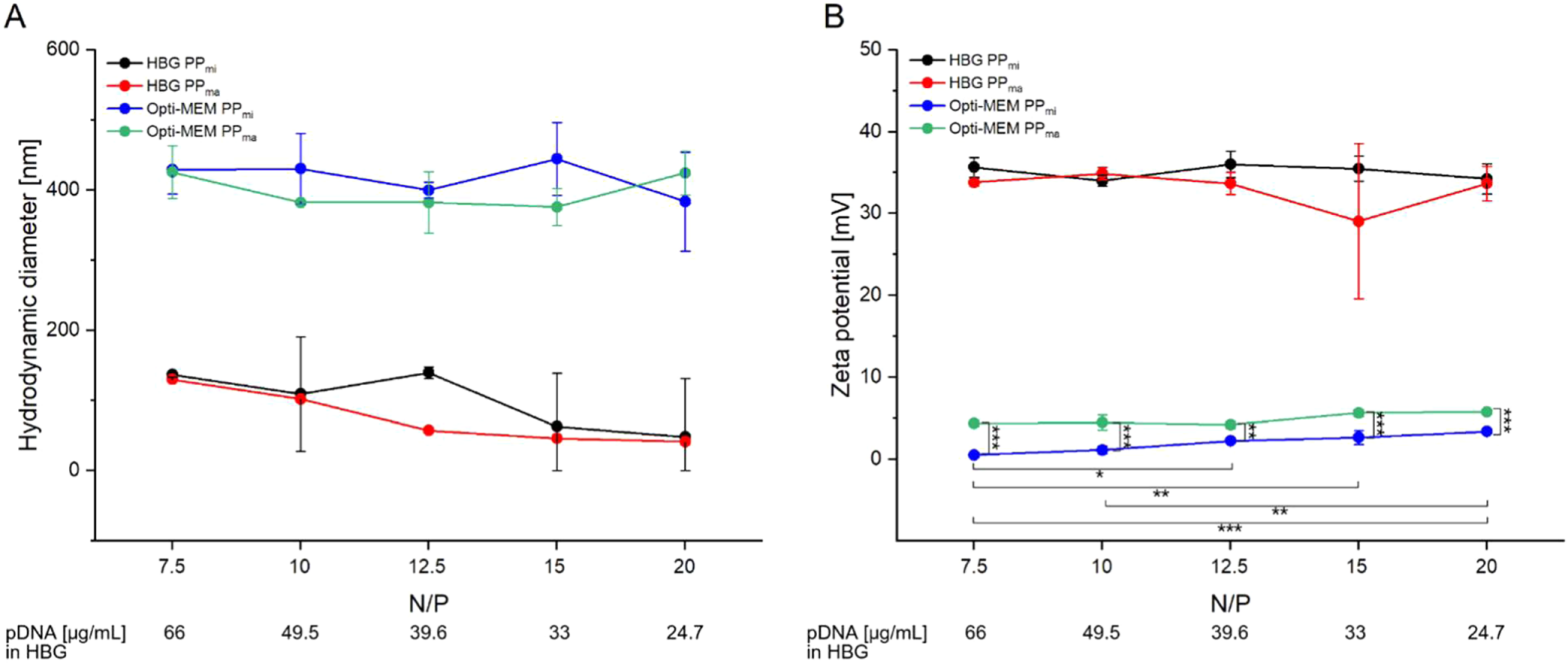
Hydrodynamic diameter (A) and zeta potential (B) of microfluidic (Setup B “mi”; PP_mi_) and manual (PP_ma_) produced polyplexes at different N/P ratios after 20 min incubation in HBG buffer (complexation step; HBG PP_mi_, HBG PP_ma_) and 10 min incubation in Opti-MEM (dilution step; Opti-MEM PP_mi_, Opti-MEM PP_ma_, dilution factor: 1/10). Polymer concentration fixed at 64 μg/mL in HBG buffer corresponding to 6.4 μg/mL after dilution in Opti-MEM, DNA concentration as indicated. Lines serve as guides to the eye. Data represents mean values ± SD with *n* = 3. * *p* < = 0.05; ** *p* < = 0.01; *** *p* < = 0.001.

As the N/P ratio increased from 7.5 to 20, the size of the microfluidic-generated polyplexes was observed to change from 137 to 48 nm (complexation in HBG buffer, Figure 6A). The manually produced polyplexes showed a comparable dependency on the N/P ratio with values of 130 nm at the lowest and 41 nm at the highest N/P ratio. The size of the microfluidic polyplexes at an N/P ratio of 12.5 was deemed to be a nonstatistically significant outlier. Overall, no statically significant differences in size were observed between PP_ma_ and PP_mi_ for the investigated N/P ratios and also not between the N/P ratios. The small size of the polyplexes at N/P 20 is in line with our previously published results.^81^ HBG is a low-ionic-strength matrix, resulting in minimal charge shielding. This allows for strong attractive interactions between the oppositely charged pDNA and l-PEI molecules within the polyplexes and significant electrostatic repulsion between the individual charged polyplexes - ultimately leading to the formation of small polyplexes.

Upon the 10-fold dilution of the preformed polyplexes in Opti-MEM, a size increase was observed to 376–426 nm for PP_ma_ and 384–445 nm for PP_mi_ (i.e., differences between N/P ratios and complexation methods were not statistically significant). These results are in line with previously published ones, where l-PEI-base polyplexes were said to have hydrodynamic sizes between 350 and 430 nm after complexation in HBS buffer and dilution in Opti-MEM using conventional preparation methods.^71^ This can be ascribed to the increase in ionic strength during the dilution step, where NaCl primarily acts as a charge shield. This weakens electrostatic interactions, likely contributing to polyplexes swelling due to charge screening of the stabilizing outer shell, as well as aggregation caused by colloidal instability.^31^ Hu et al. have recently demonstrated that the size of pDNA/PEI complexes plays a critical role in achieving high transfection efficiency in H293F cells, identifying 400–500 nm as the optimal size range.^82^ The polyplexes produced here thus fall within this optimal range, making them promising tools for the efficient transfection of mammalian cell with l-PEI.

By contrast, most of the published PEI-based transfection protocols for ARPE-19 cells used a conventional polyplex preparation method that does not include a dilution step following the 20 min incubation of polyplexes in a buffered solution. Consequently, it can be speculated that the resulting polyplexes remained small, potentially making them less suitable for achieving high transfection efficiency. We speculated that this may have contributed to the less satisfactory results found in the pertinent literature on PEI-based transfection of ARPE 19 cells. Incidentally, as demonstrated in the transfection optimization study (Figure 4), PP_ma_ results in high transfection efficiency in ARPE-19, which may be linked to their size as measured by DLS.

The Zeta potential measured for PP_mi_ (+34–36 mV) and PP_ma_ (+29–34 mV) after complexation in the HBG buffer was comparable across all N/P ratios, with minor differences of no statistical significance observed between the production ways and N/P ratios (Figure 6B). These results are consistent with the gel retardation assay analysis (Figure S4) and demonstrate full charge compensation of pDNA above an N/P ratio of 5. Diluting the preformed polyplexes in Opti-MEM led to a significant (approximately 6-fold) reduction in zeta potential. PP_mi_ exhibited a charge ranging from +0.5 to +3.4 mV, while that of PP_ma_ was between +4.2 and +5.7 mV, the latter consistent with previously published data from our group.^71^ The statistically significant differences between the two preparations and also between the different N/P ratios for the microfluidic samples could be due to the more homogeneous mixing achieved during microfluidic production.

In an effort to move toward a more fully automated polyplex preparation, we also automated the dilution step in Opti-MEM following the complexation step in HBG. Since our setup requires that the two solutions (polyplex in HBG and Opti-MEM) be pumped simultaneously using a single syringe pump, the ratio of the syringe diameters is important for achieving the desired 1:10 dilution. Based solely on the available syringes, the dilution ratio with Opti-MEM could not be exactly set at 1:10, and therefore the concentrations of pDNA and PEI solutions in the upstream complexation step had to be adjusted (Setup B “mi_adj._”) in order to ensure the final polyplex concentration was appropriate. Moreover, to maintain the correct Opti-MEM-to-polyplex ratio in the Chip_dilution_ (Figure 1B), the channel width and height in the channel for Opti-MEM were also adjusted, increasing the dead volume to 63.5 μL. As above, the N/P ratio was set to 10 and a flow rate of 2 mL/min was used for the complexation step (Chip_complexation_, Figure 1A). The preformed polyplexes were then transferred at a flow rate of 1.2 mL/min into the Chip_dilution_ unit, where they were mixed with Opti-MEM. Polyplexes prepared using this approach (Setup C “semi-automated”) are referred to as PP_semi-automated_. For comparison, a sample was similarly prepared except that only the complexation step was performed microfluidically, while the dilution step was carried out manually. This sample is referred to as PP_mi adj._ Additionally, PP_mi_, as prepared in Figure 6 were also included for the sake of comparison.

The hydrodynamic diameter, zeta potential and polydispersity index of polyplexes were determined at the end of the complexation step (after 20 min) in HBG buffer, and following dilution and 10 min incubation in Opti-MEM (see Supporting Information Figures S8 and S9). Independently of the preparation methods, all polyplexes were found to have a size of 140 to 152 nm after incubation in HBG and of 367 to 439 nm after dilution in Opti-MEM without any significant differences, which is consistent with the values reported in Figure 6A. In terms of the zeta potential, values measured after incubation in HBG were also found to be comparable with those measured previously and given in Figure 6B, i.e., ≈+32 mV. However, significant and potentially detrimental differences in the Zeta potential were observed following the dilution step in Opti-MEM; whereas PP_mi_ had a Zeta potential comparable to that measured previously in Figure 6B, PP_mi adj._ and PP_semi-automated_ showed negative Zeta potential values of −4.4 mV and −2.2 mV, respectively.

In comparison to PP_mi_, both the PP_mi adj._ and PP_semi-automated_ polyplexes were generated using adjusted (reduced) concentrations of pDNA and l-PEI in the complexation step. As a result, the polyplex concentration in the subsequent dilution step is also lower. The reasons underlying changes in charge remain speculative; however, one possible explanation could be that a lower polyplex concentration in the dilution step with Opti-MEM allows for different molecular interactions compared to higher concentrations. This may enable more efficient and uniform protein adsorption, potentially leading to changes in the surface charge. As a result, the zeta potential of the diluted polyplexes (PP_mi adj._ and PP_semi-automated_) could shift to a negative value, likely due to the formation of a negatively charged protein corona. Opti-MEM contains proteins like insulin (pI 5.3) and transferrin (pI 5.2–5.6), which are negatively charged at physiological pH (∼7) and can electrostatically interact with polyplexes prepared in HBG buffer, thereby altering their surface charge. Volpatti et al. (2021) have previously showed that insulin associates with l-PEI, potentially contributing to charge modification.^83^

In contrast, when polyplexes are prepared in the microfluidic chamber at higher concentrations of pDNA and l-PEI (PP_mi_), protein adsorption may be less efficient due to higher polyplex density, steric hindrance, or limited protein availability per particle, leading to only a partial decrease in zeta potential without full charge reversal.

As there was also a significant difference between the polyplexes diluted microfluidically (PP_semi-automated_) and manually (PP_mi adj._) with Opti-MEM, the type of dilution also seems to matter. The role of the micromixer in facilitating these interactions still remains to be elucidated in future experiments; for present purpose, we will only hypothesize that the microfluidic mixer enables faster, more uniform, and efficient protein adsorption onto the polyplexes, thereby leading to charge reversal. By contrast, manual vortexing is more chaotic, and might result in inhomogeneous mixing- thus, leading to less efficient or incomplete protein adsorption and no charge reversal.

### Comparison of Transfection Outcomes Using Microfluidic-Based Polyplexes Generation

As shown in Figures 5 and 6, polyplexes with physicochemical properties comparable to those produced by conventional manual mixing techniques can be successfully generated using the developed microfluidic system. The next step was to assess their efficiency in transfecting ARPE-19 cells. Initially, we evaluated the impact of using the Chip_complexation_ microfluidic system to complex pDNA and polymer in HBG buffer, while the dilution step with Opti-MEM was still performed manually (Setup B “mi”; PP_mi_). This was done because automating both the complexation and dilution steps was shown to slightly influence the net charge of the polyplexes (Figure S8D), a parameter that may also affect transfection outcomes. The N/P ratio was adjusted by varying the amount of pDNA while polymer density (40 μg polymer per 10^6^ cells) and polymer concentration (6.4 μg/mL during transfection) were kept constant. For the sake of comparison, corresponding polyplexes were once again prepared manually (Setup A “ma”; PP_ma_). Transfection was performed in 12-well plates using 0.5 mL of transfection mixture, and transfection efficiency along with cell viability were measured 48 h post-transfection. Notably, to assess interlaboratory variability, freshly prepared pDNA and l-PEI stock solutions were used for this set of experiments, following the same preparation protocol as in Figures 3 and 4. In addition, the transfection was performed by a different operator. The results are summarized in Figure 7.

**Figure 7:**
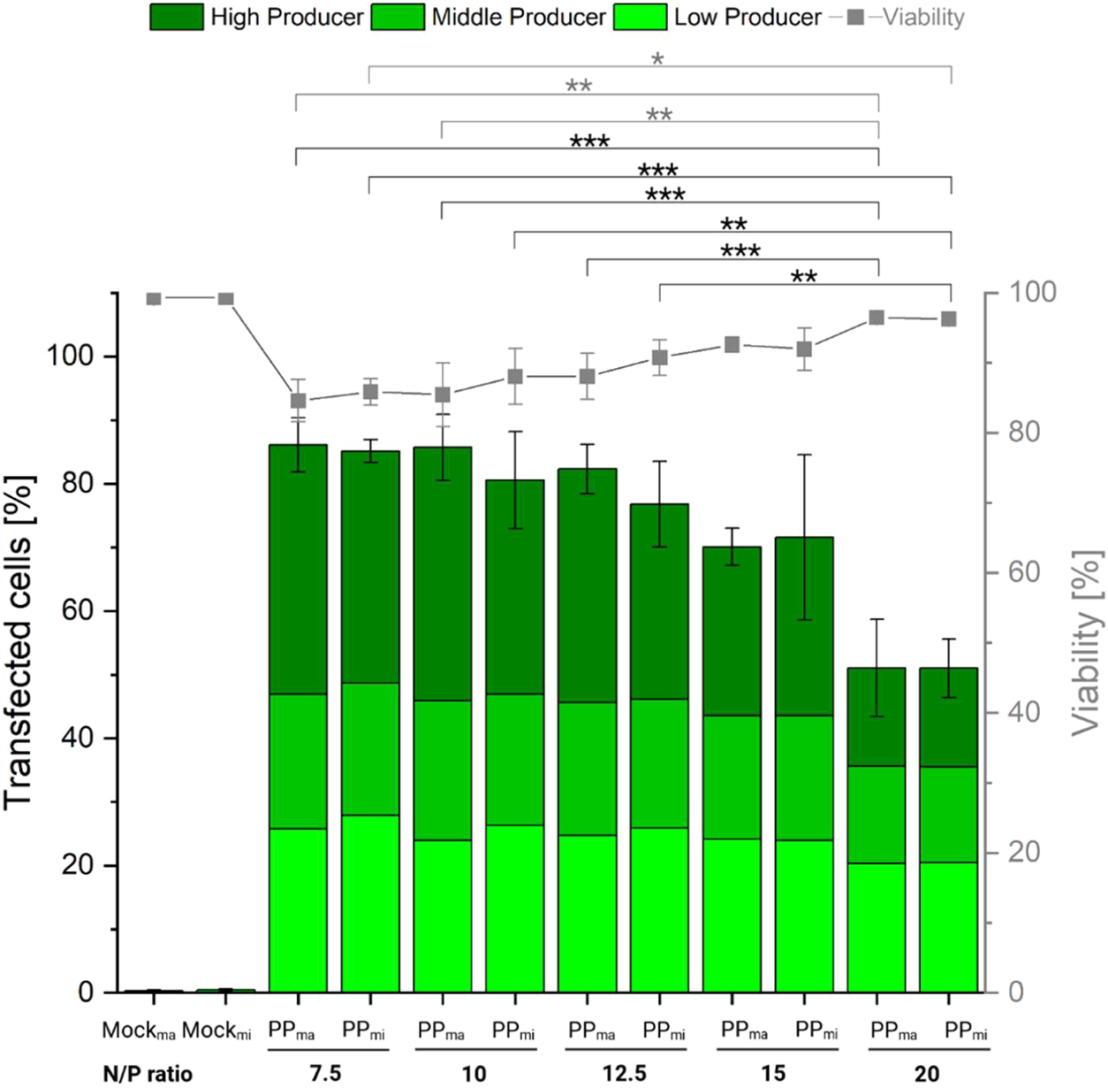
Comparison of transfection efficiency and cell viability depending on manual (Setup A “ma”; Mock_ma_, PP_ma_) and microfluidic (Setup B “mi”; Mock_mi_, PP_mi_) produced polyplexes and different N/P ratios. Total cells: 8 × 10^4^ per well, 12-well plate, transfection volume 0.5 mL (0.05 mL polyplex solution), fix polymer density (40 μg polymer per 10^6^ cells) and polymer concentration (6.4 μg/mL during transfection), N/P ratio adjusted by pDNA amount, contact time 2 h, recovery time post-transfection: 48 h. Lines are guides to the eye. Data represents mean values ± SD with *n* = 3. Detailed statistical analytics are shown in Figure S10. Of note, freshly prepared pDNA and l-PEI stock solutions were used for the PP_ma_-based transfection, following the same preparation protocol as in Figures 3 and 4. The transfection was performed by a different operator.

Regardless of the method used to prepare the polyplexes, both TE and cell viability were found to be comparable. TE reached 77–86% at N/P ratios from 7.5 to 12.5. However, increasing the N/P ratio beyond this point resulted in a decline in TE, which became statistically significant at an N/P ratio of 20 (Figure S10). This reduction may be due to the decreasing amount of pDNA available as the N/P ratio increases, resulting in fewer or no pDNA molecules being delivered to the cells during transfection.

The proportion of high producers (based on eGFP expression levels) showed a negative correlation with the N/P ratio. Specifically, as the N/P ratio increased, the fraction of high producers declined, with this effect becoming more pronounced at N/P ratios ≥ 15. This trend may be attributed to the reduced availability of pDNA molecules in the transfection mixture, which likely results in lower pDNA uptake per cell, as hypothesized earlier. Importantly, the method used to prepare polyplexes (manual vs automated) did not seem to influence the overall level of gene expression. Cell viabilities remained consistently ≥ 83%, although a significant trend was only observed between NP ratio 7.5 (and 10 only PP_ma_) and 20, indicating slightly higher viability at higher N/P ratios, which could correlate with a reduction in the proportion of high producers (Figure S11). This observation aligns with the hypothesis proposed earlier in this study and suggests a potential link between eGFP cytotoxicity and cellular viability.

The transfection outcomes achieved with automatically prepared polyplexes are comparable to those obtained through manual preparation, with no significant improvement in efficiency. However, the automated setup is expected to help minimizing interlaboratory experimental variation - even for well-trained operators- while offering a notable advantage for less-experienced users by enabling consistent transfection efficiency without the need for extensive training, which is typically required for manual polyplex preparation.

In the next step, both complexation in HBG buffer and dilution with Opti-MEM were performed using HC micromixers (Setup C “semi-automated”; PP_semi-automated_). For the sake of comparison, PP_mi_ (Setup B “mi”) and PP_mi adj._ (Setup B “mi_adj._”) were also included. In all cases, the final polymer concentration and polymer density applied to the cells were identical to those used in Figure 7. Since we previously demonstrated that the N/P ratio does not significantly impact results within the range of 7.5 to 12.5, we limited our testing to N/P ratio 10. The results are shown in Figure 8.

**Figure 8:**
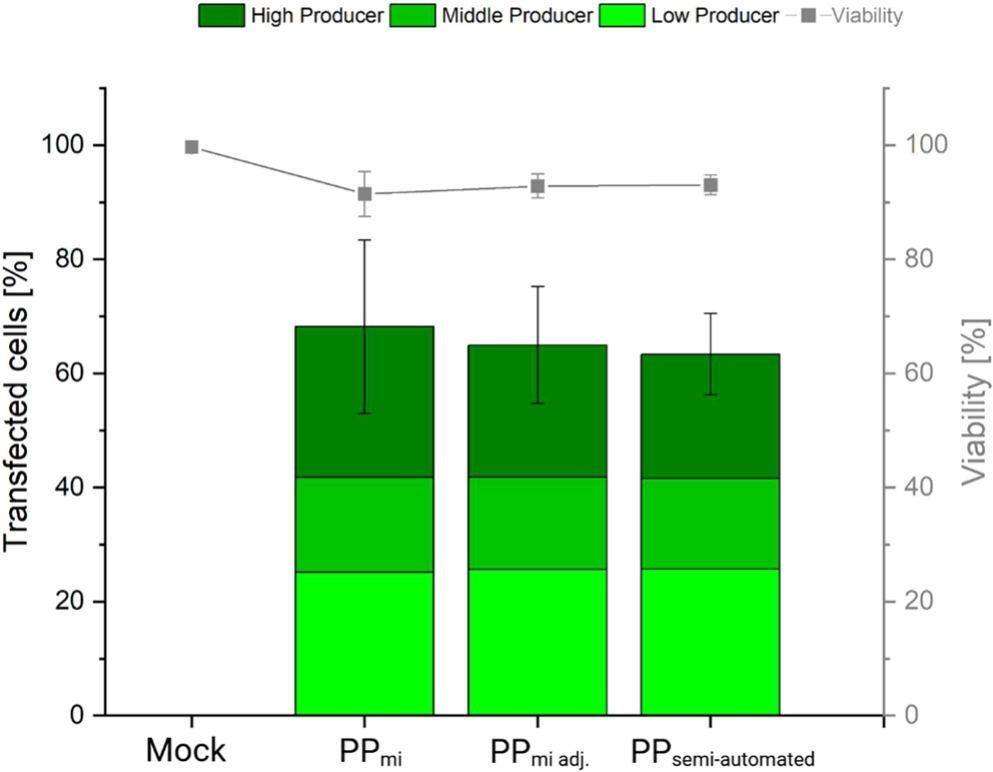
Influence of the microfluidic methods used to prepare polyplexes on TE and cell viability. Total cells: 8 × 10^4^ per well, 12-well plate, N/P ratio: 10, transfection volume 0.5 mL (0.05 mL polyplex solution), polymer density 40 μg per 10^6^ cells, polymer concentration 6.4 μg/mL during transfection, contact time 2 h, recovery time post-transfection: 48 h. Lines are guides to the eye. “PP_mi_”: Microfluidic-based complexation step (Setup B “mi”) with manual-based dilution step; “PP_mi adj._”: Microfluidic-based complexation step (Setup B “mi_adj._”) with manual-based dilution step, using adjusted pDNA and PEI start concentrations; “PP_semi‑automated_” (Setup C “semi-automated”): Microfluidic-based complexation and dilution steps with adjusted pDNA and PEI start concentrations. Data represents mean values ± SD with *n* = 3. Full statistical analysis see Figure S12.

Overall, no significant differences were observed between the transfections performed with the various polyplex preparations. TE averaged 66%, while cell viability was consistently ≥ 90%. Furthermore, these results are comparable to those achieved using the manual method for polyplex preparation, which yielded a TE of 86% and a cell viability of 85% (Figure 7). The slight decrease in TE compared to the results shown in Figure 7 may be attributed to the use of cells at a higher passage number; passage number has been shown to significantly impact transfection efficiency in ARPE-19 cells, particularly when Lipofectamine transfection reagent is used.^21^ In addition, transfection with the semi-automated approach (PP_semi-automated_) takes a little longer, since the syringes for the complexation and dilution step still have to be drawn up manually, which extends the incubation times somewhat. These samples were taken as a time reference for the others to ensure comparability between the samples. The slightly longer times for complexation and dilution could also have led to lower TE results. In future work, these steps should be automated in order to be able to adhere to the exact times and come closer to a full automated transfection system.

Mock transfections always display a cell viability ≥ 98%, showing that preparation of polyplexes in a microfluidic system has no effect on the cell viability and thus validating that the resin used to produce the micromixer and the residence time of the polyplexes in the microfluidic system did not lead to accumulation of leachables that could negatively influences cell viability.

These results demonstrate that high-quality polyplexes (suitable for transfection) can be produced in a semi-automated fashion within 3D-printed microfluidic systems featuring integrated HC micromixers. The transfection parameters established and optimized manually could be effortlessly applied to transfect ARPE-19 cells with polyplexes that are prepared using a microfluidic approach. Since no significant differences in performance were observed, the microfluidic method offers the key advantage of automation, simultaneously reducing experimenter-induced variability while also providing numerous other benefits over the conventional manual approach. This automation paves the way for a fully automated transfection process, further reducing variability and enabling scalable polyplex production.

## Conclusion

In this study, we successfully optimized a 25 kDa l-PEI-based transfection protocol for ARPE-19 cells, achieving a transfection efficiency (TE) of 66 to 89% and cell viability of around 80%. Key optimizations included adjusting the N/P ratio by varying the pDNA instead of increasing the l-PEI concentration, as well as reducing the contact time between cells and polyplexes, both of which minimize cell mortality. Additionally, reducing the transfection volume was observed to improve transfection outcomes by enhancing the likelihood of interactions between cells and polyplexes and we demonstrated the successful transfer of the optimized protocol to a semi-automated 3D-printed microfluidic system for polyplexes preparation. This approach enabled the controlled and reproducible production of polyplexes without significant losses in TE or changes in viability, even though only minimal differences in physicochemical properties (i.e., size and charge) were observed between manually and microfluidically produced polyplexes. While microfluidically produced polyplexes may not necessarily yield superior transfection outcomes, they do offer a consistency, thereby promising to reduce experimental variability as the polyplex production is taken over by the syringe pump and the microfluidic chip and no longer by the experimenter. In addition, the ability to adjust polyplex size through changes in flow rate further enhances the flexibility of the method. It is worth noting that even experienced operators can encounter variability in transfection outcomes when using the manual mixing method due to the uncontrolled formation of polyplexes. Our microfluidic system enables the preparation of polyplexes in a much more standardized manner. This enhanced consistency is expected to be particularly useful for less experienced operators, since it should help them to generate high-quality polyplexes from scratch and thus focus on refining the transfection process itself. Furthermore, this microfluidic system is specifically designed to streamline the automation of polyplex production while also simultaneously enabling scalable and standardized production and maintaining the stringent quality control standards required for *in vivo* use. This system thus offers a significant advantage over others due to its scalability. It can be used in small-scale applications such as laboratory or testing phases and scaled up through parallelization and increased flow rates for larger-scale operations. Additionally, the production via 3D printing with autoclavable material allows for rapid design customization, grants researchers the ability to print standard connectors to prevent leakages, and facilitates sterile integration into processes.^43,45,62–64^

In summary the optimized transfection methods presented in this paper outperforms previously published results, and (due to its reliance on l-PEI) it also offers a broadly applicable solution for researchers worldwide. Moreover, its potential for translational research and adaptability for *in vivo* applications adds to its relevance with respect to gene-based therapies, including for age-related macular degeneration. Notably, l-PEI is (at least to our knowledge) the only transfection agent available in GMP-grade quality, a critical factor for the future translation of this protocol into clinical applications. We believe that these reported results will accordingly lay the groundwork for future research on ARPE-19 and primary RPE cells, hopefully helping to facilitate the development of effective gene-based therapies for retinal diseases.

## Supplementary Material



## Notes

Supplementary method: MTT assay; Supplementary table: Table S1: Parameters used for DLS measurements; Supplementary figures: Figure S1: Picture of the setups of the two microfluidic systems for polyplex formation and dilution; Figure S2: Flow cytometry analysis of transfected ARPE-19 cells: Representative plots and gating strategy; Figure S3: MTT assay; Figure S4: Gel retardation analysis of polyplexes at different N/P ratios; Figure S5: Relationship between the incidence of “high producers” and “viability”, based on the data presented in Figure 4; Figure S6: Polydispersity index of the polyplexes in HBG buffer during the initial complexation step in microcentrifuge tubes; Figure S7: Polydispersity index of microfluidic- and manual-produced polyplexes at different N/P ratios; Figure S8: Hydrodynamic diameter and zeta potential for different microfluidic produces polyplexes with a N/P of 10; Figure S9: Comparison of polydispersity index of microfluidic-produced polyplexes at a N/P ratio 10; Figure S10: Statistical analysis for comparison of transfection efficiency (A) and cell viability (B) depending on manual and microfluidic produced polyplexes and different N/P ratios; Figure S11: Relationship between the incidence of “high producers” and “viability” 48 h post-transfection, based on the data presented in Figure 7; Figure S12: Statistical analysis for comparison of transfection efficiency (A) and cell viability (B) depending on different microfluidic produced polyplexes at N/P ratio of 10 (PDF)

## ⊥

Physiology, Institute of Theoretical Medicine, University of Augsburg, Augsburg 86159, Germany

## Abbreviations

AAV: Adeno-Associated Virus
AMD: age-related macular degeneration
AO: Acridine Orange
ARPE-19 cell line: immortalized retinal pigmented epithelial cells
ATMP: Advanced Therapy Medicinal Product
CHO_sus_: Chinese Hamster Ovary cells in suspension
CRALBP: cellular retinaldehyde-binding protein
DLS: dynamic light scattering
DMEM: Dulbecco’s Modified Eagle’s Medium
DPBS: Dulbecco’s phosphate-buffered saline
eGFP: enhanced Green Fluorescent Protein
FSC: forward scatter
HBG: buffer containing 20 mM Hepes, 5 wt % glucose, pH 5.5
FCS: fetal calf serum
FDM: fused deposition modeling
l-PEI: linear polyethylenimine
ma: manual
MEM: Minimal Essential Medium
mi: microfluidic
Mock: negative control for transfection without pDNA and PEI
mi_adj_.: microfluidic with adjusted concentrations
N: concentration (mM) of nitrogen residues in l-PEI
p: nmoles phosphate in pDNA
pDNA: plasmid DNA
Pol. conc.: polymer concentration
Pol. dens.: polymer density
PI: Propidium Iodide
PP: polyplex
RPE: retinal pigment epithelium
SAW: surface acoustic wave
SD: standard deviation
SLA: stereolithography
SSC: side scatter
TE: transfection efficiency
UV: ultraviolet
7-AAD: 7-Aminoactinomycin
