# Supplementary material for: High-Efficiency l‑PEI-Based Transfection of ARPE-19 Cells Using a Multiparametric Approach and Automated Polyplex Formation with a 3D-Printed Microfluidic System
Source: Chem Bio Eng. 2025 Sep 10;2(12):695–710. doi: 10.1021/cbe.5c00059 (PMC12746001; doi:10.1021/cbe.5c00059)
Supplement: Supplementary file 1 [file be5c00059_si_001.pdf]

Supporting Information

High-Efficiency l-PEI-Based Transfection of ARPE-19 Cells Using a Multiparametric Approach and Automated Polyplex Formation with a 3D-Printed Microfluidic System

Daniel Keim[1,‡], Michaela Dehne[2,3,‡], Patricia Miller[2†], Valérie Jérôme[1], Janina Bahnemann[2,4,] and Ruth Freitag[1*]*

[1] Process Biotechnology, University of Bayreuth, Bayreuth, 95444 Universitätstraße 30, Germany

[2] Technical Biology, Institute of Physics, University of Augsburg, Augsburg, Universitätsstraße. 1 86159, Germany

[3] Institute of Technical Chemistry, Leibniz University Hannover, Hannover, Callinstraße 5 30167, Germany

[4] Centre for Advanced Analytics and Predictive Sciences (CAAPS), University of Augsburg, Augsburg Universitätsstraße 6 86159 Germany

*ruth.freitag@uni-bayreuth.de *janina.bahnemann@uni-a.de

‡ These authors contributed equally.

Authors ORCID

Daniel Keim: 0000-0002-8338-8041

Michaela Dehne: 0009-0001-6015-1084

Patricia Miller: 0009-0002-3899-9211

Valérie Jérôme: 0000-0001-6492-2168

Janina Bahnemann: 0000-0002-7008-1673

Ruth Freitag: 0000-0002-6569-9137

Supplementary methods

MTT Assay

The *in vitro* cytotoxicity of 25 kDa L-PEI was evaluated using the MTT assay following the ISO 10993-5 protocol. Briefly, ARPE-19 cells were seeded at a density of 1×10^4 cells per well in 96-well plates and incubated for 24 hours. After incubation, the culture medium was aspirated and replaced with 100 µL of freshly prepared polymer dilutions in growth medium at concentrations ranging from 0.001 to 0.08 mg/mL. Cells were then incubated for an additional 24 hours. Following the incubation period, the medium was removed and replaced with 50 µL of freshly prepared MTT reagent (1 mg/mL MTT in MEM without phenol red, sterile filtered). The cells were incubated for 2 hours, after which the supernatant was discarded. To dissolve the formazan crystals, 100 µL of isopropanol was added to each well, and the plate was shaken at 600 rpm for 5 minutes. Absorbance was measured at 570 nm (reference wavelength 650 nm) using a Tecan GENios Pro plate reader (Tecan Austria GmbH, Grödig, Austria).

Untreated cells, maintained under identical conditions, served as the negative control (blank, corresponding to 100 % viability). Cells treated with 0.3 % v/v Triton X-100 were used as the positive control. Independent experiments were conducted with six replicates.

Cell viability (%) was calculated according to equation (Eq1):

$$Viability\ [\%] = \frac{Abs570_{\text{sample}}}{Abs570_{\text{blank}}} x\ 100 \qquad \text{(Eq1)}$$

where: $Abs570_{sample}$ is the mean value of the measured absorption of the test sample; $Abs570_{blank}$ is the mean value of the measured absorption of the negative control. The lethal dose (LD_{50}) was defined as the polymer concentration at which 50 % cell viability was observed.

Calculations

To estimate the number of polymer chains per cell, the following calculation was performed:

$$m_{\text{cell}} = \frac{60 \times 10^{-6}\ \text{g}}{10^{6}\ \text{cells}} = 6 \times 10^{-11}\ \text{g/cell}$$

$$n_{\text{PEI}} = \frac{m_{\text{cell}}}{M_{\text{PEI}}} = \frac{6 \times 10^{-11}\ \text{g}}{2.5 \times 10^{4}\ \text{g/mol}} = 2.4 \times 10^{-15}\ \text{mol}$$

$$N_{\text{chains}} = n_{\text{PEI}} \times N_A = (2.4 \times 10^{-15}) \times (6.022 \times 10^{23}) \approx 1.4 \times 10^{9}$$

Where m_{cell} is the mass of a single cell, n_{PEI} is the number of moles of polymer added to the cells during transfection, M_{PEI} is the molecular weight of PEI, and N_{chains} is the total number of polymer chains present in the well during transfection.

Supplementary Tables

Table S1: Parameters used for DLS measurements

Measurement cell	Univette Low Volume
Module Type	BM10
Target temperature	20 ˚C
Solvent Name	10mM HEPES
Refractive index	1,330902642436
Viscosity	1,002256 mPa.s
Relative permittivity	80,0644498798748
**Hydrodynamic diameter**	
Measurement angle	Automatic
Equilibration time	30 s
Max. number of runs	20 (series measurement) / 60 (single measurement)
Measurement time	5 s (series measurement) / 10 s (single measurement)
Material Name	Protein
Refractive index	1.4500
Absorption coefficient	0.0010 1/m
**Zeta potential**	
Equilibration time	2 min
Approximation	Smoluchowski
Henry factor	1,5
Adjustment mode	Automatic

Maximal voltage	40 V
Quality Mode	Manual
Max. number of runs	100

Supplementary figures

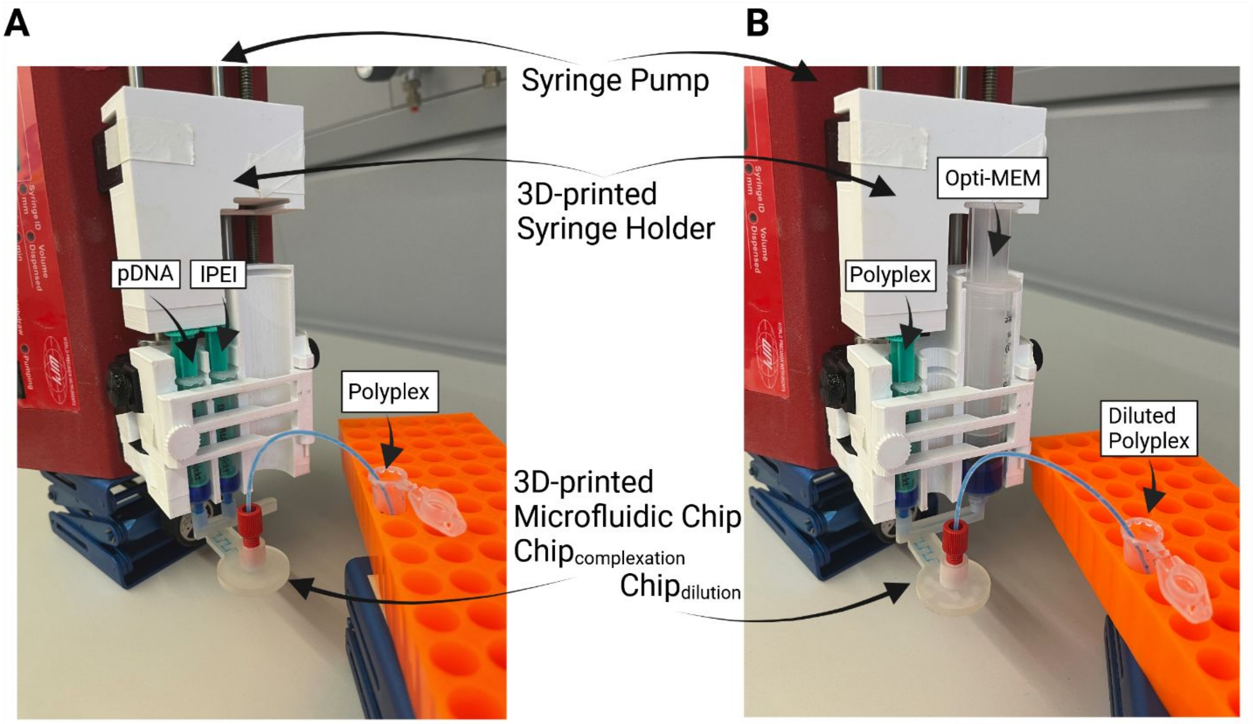

Figure S1. Picture of the setups of the two microfluidic systems for polyplex formation and dilution. A: System for producing the polyplexes in HBG buffer ($Chip_{complexation}$). B: System for mixing the pre-formed polyplexes with Opti-MEM during the dilution step ($Chip_{dilution}$). For better visibility of the liquids and channels, a solution with a blue dye (bromphenol blue) was used.

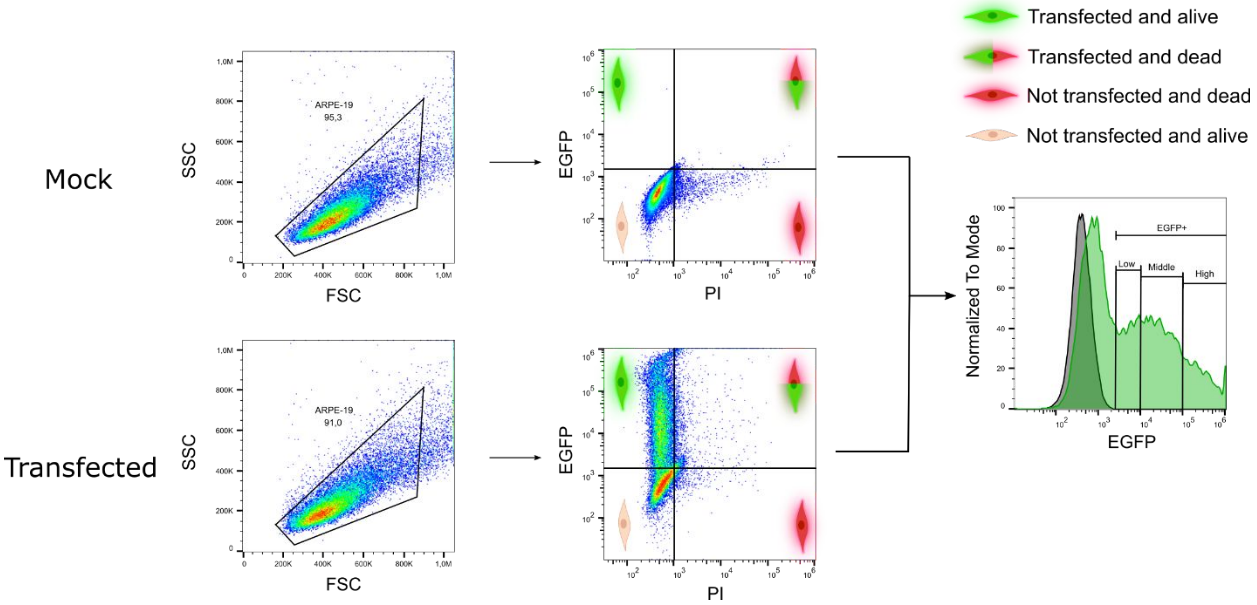

Figure S2. Flow cytometry analysis of transfected ARPE-19 cells: Representative plots and gating strategy. Flow cytometry analysis was performed to evaluate transfection efficiency and cell viability in ARPE-19 cells. Mock-transfected cells were used to set the measurement parameters. First, single, non-apoptotic cells based on forward and side scatter (FSC/SSC) properties were selected. This approach excluded dead cells, debris, and cellular aggregates, defining a region labeled as gate "ARPE-19." To evaluate both transfection efficiency and post-transfection viability, the gated "ARPE-19" population was further analyzed using a two-parameter dot plot to assess EGFP expression (EGFP, log scale) and viability (PI, log scale). To determine the distribution of transgene expression levels, fluorescence intensity data (EGFP, log scale) was represented in a histogram plot. Within the gated "ARPE-19" population, three expression levels were identified: low producers (fluorescence intensity between 10^3-10^4 (2 x 10^4-2 x 10^5), "Low"), middle producers (fluorescence intensity between 10^4-10^5 (2 x 10^5-2 x 10^6), "Middle"), and high producers (fluorescence intensity greater than >10^5 (>2 x 10^6), "High").

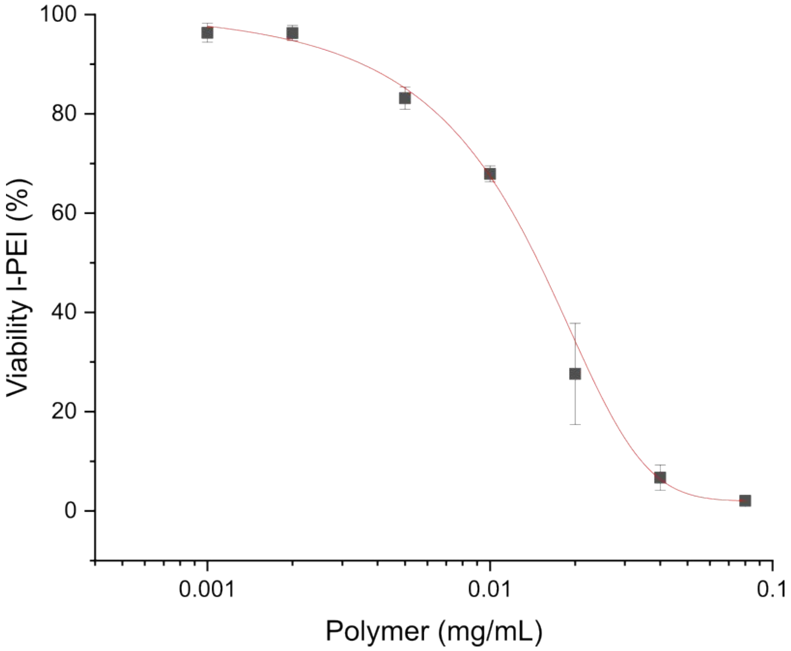

Figure S3. Cell viability as function of 25 kDa l-PEI concentration determined by MTT assay in ARPE-19 cells. Data represent mean ± SD, n ≥ 6. Calculated LD_{50}: 0.0101 ± 0.0024 mg/mL.

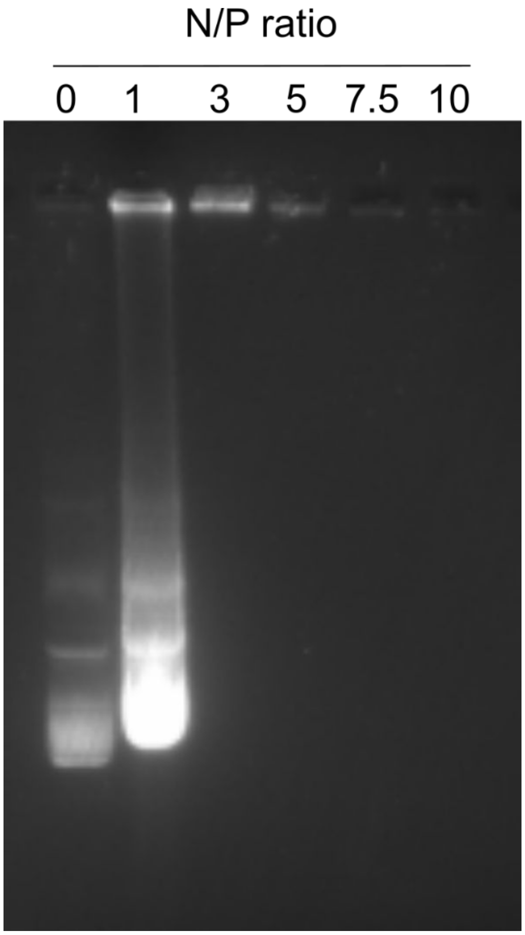

Figure S4. Gel retardation analysis of polyplexes at different N/P ratios.

Polyplexes were prepared using the manual transfection method. A fixed amount of 2 μg of pEGFP-N1 plasmid was combined with increasing amount of l-PEI to achieve the desired N/P ratio. Following complexation, the electrophoretic mobility of the polyplexes was analyzed on a 1 % agarose gel. The image shows the gel retardation of the polyplexes as a function of the N/P ratio, with the specific ratios indicated above each lane.

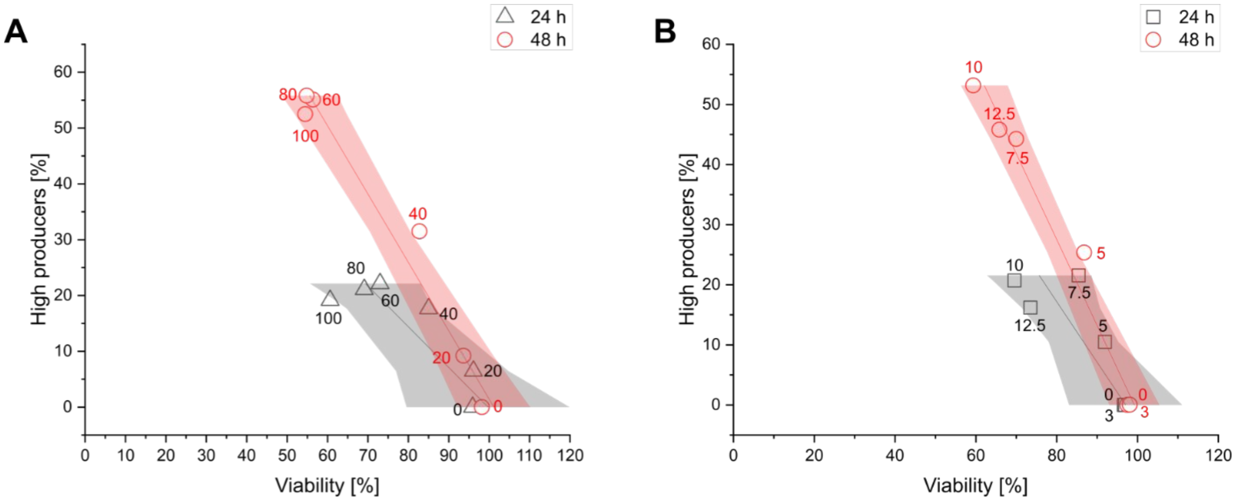

Figure S5. Relationship between the incidence of "high producers" and "viability", based on the data presented in Fig. 4.

A: Effect polymer density (Fig. 4A), (24 h post-transfection) y= -1.4 x + 99.7 R^2: 0.640; (48 h post-transfection) y= -0.82 x + 101.2 R^2: 0.950

B: Effect NP ratio (Fig. 4B), (24 h post-transfection) y= -0.99 x + 97.1 R^2: 0.590; (48 h post-transfection) y= -0.70 x + 99.2 R^2: 0.960.

Red and grey lines represent the fit with a 95 % confidence band. The correlation becomes more apparent after 48 hours.

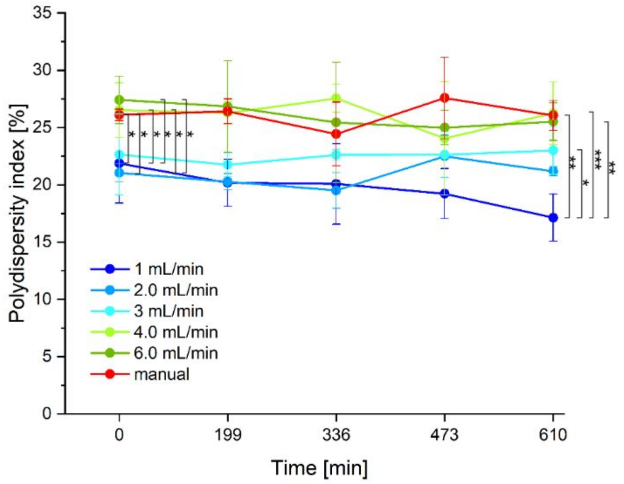

Figure S6. Polydispersity index of polyplexes in HBG buffer during the initial complexation step in microcentrifuge tubes. Polyplexes were prepared at an N/P ratio of 10 via standard manual procedure (“manual”) and via microfluidic system (Setup B “mi”) at different flow rates. pDNA amount: 5.44 µg, l-PEI: 7.04 µg. Total volume: 110 µL. Data represents mean values ± SD with n=3. * p<=0.05; ** p<=0.01; *** p<=0.001. Data represent mean ± SD with n=3. Statistical analysis was performed using Bonferroni correction: * p ≤ 0.05; ** p ≤ 0.01; *** p ≤ 0.001.

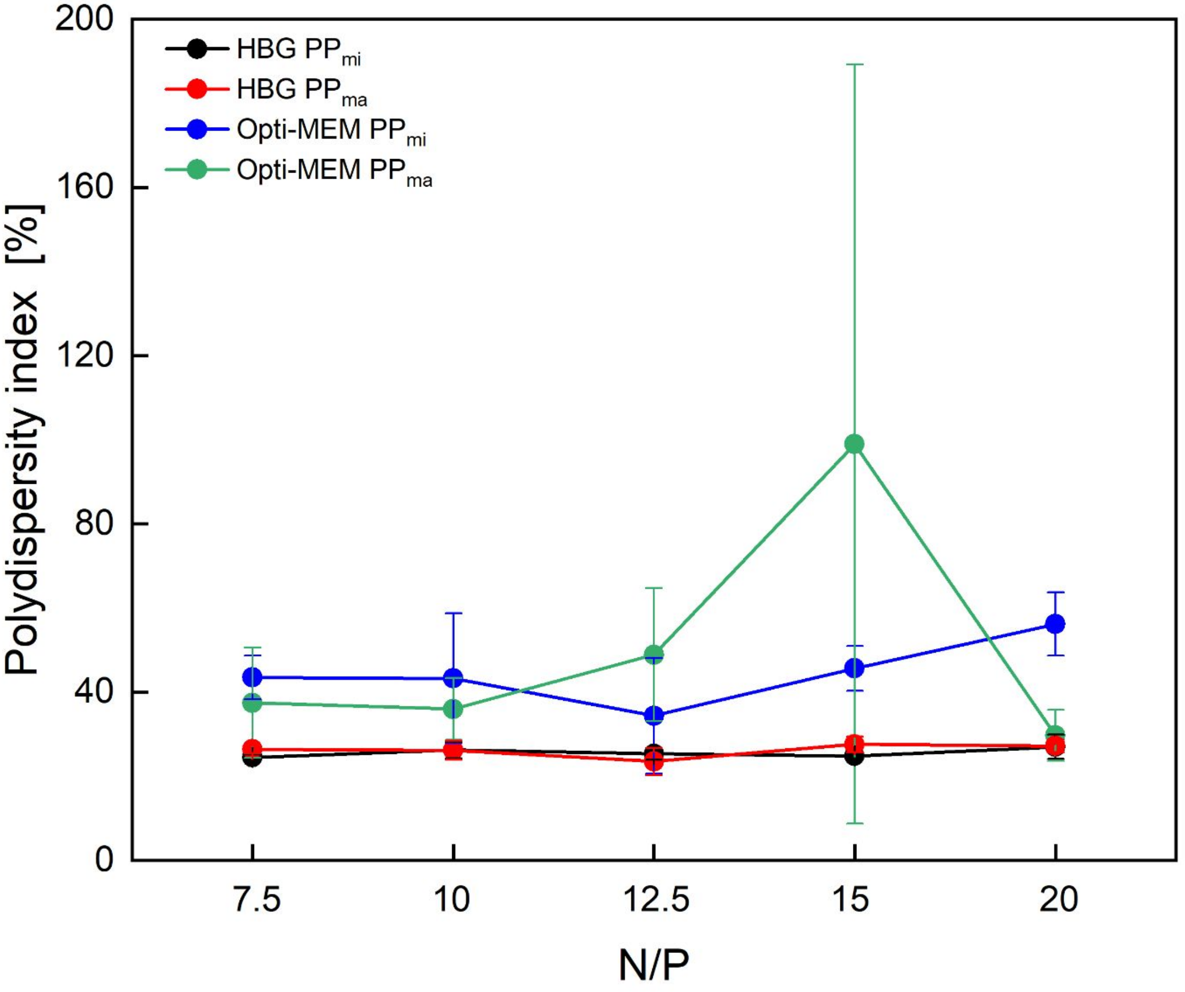

Figure S7. Polydispersity index of microfluidic- (Setup B "mi"; PP_{mi}) and manual-produced (PP_{ma}) polyplexes at different N/P ratios after 20 min incubation in HBG buffer (complexation step; HBG PP_{mi}, HBG PP_{ma}) and 10 min incubation in Opti-MEM (dilution step; Opti-MEM PP_{mi}, Opti-MEM PP_{ma}, dilution factor: 1/10). Polymer concentration fixed at 64 µg/mL in HBG buffer corresponding to 6.4 µg/mL after dilution in Opti-MEM, DNA concentration as indicated. Lines serve as guides to the eye. Data represents mean values ± SD with n=3. * p<=0.05; ** p<=0.01; *** p<=0.001

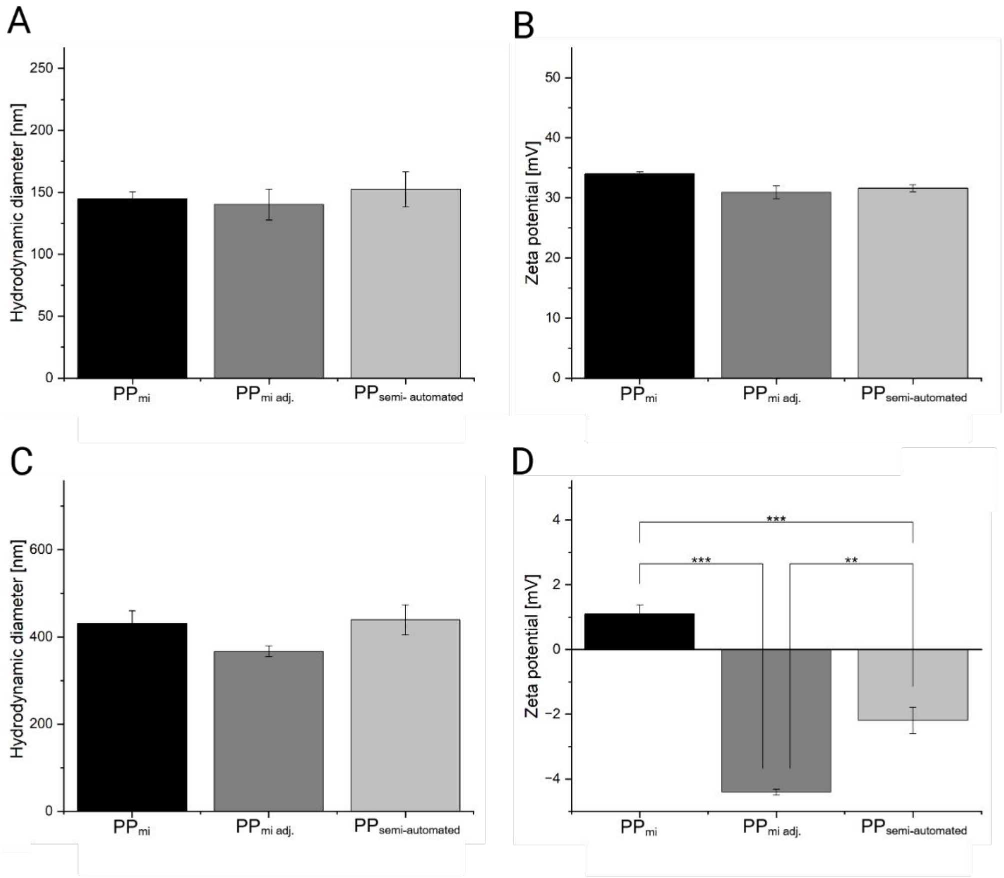

Figure S8. Comparison of microfluidic-produced polyplexes at a N/P ratio 10. The hydrodynamic diameter (A, C) and zeta potential (B, D) of microfluidic-produced polyplexes were analyzed after complexation in HBG buffer (A and B) and after dilution in Opti-MEM (B and D). PP_{mi}: microfluidic-produced polyplexes in HBG; $PP_{mi\ adj.}$: microfluidic-produced polyplexes in HBG with adjusted start concentrations; $PP_{semi\text{-}automated}$: microfluidic-produced polyplexes in HBG followed by microfluidic-dilution with Opti-MEM, using adjusted start concentrations. Data represent mean ± SD with n=3. Statistical analysis was performed using Bonferroni correction: * $p \leq 0.05$; ** $p \leq 0.01$; *** $p \leq 0.001$.

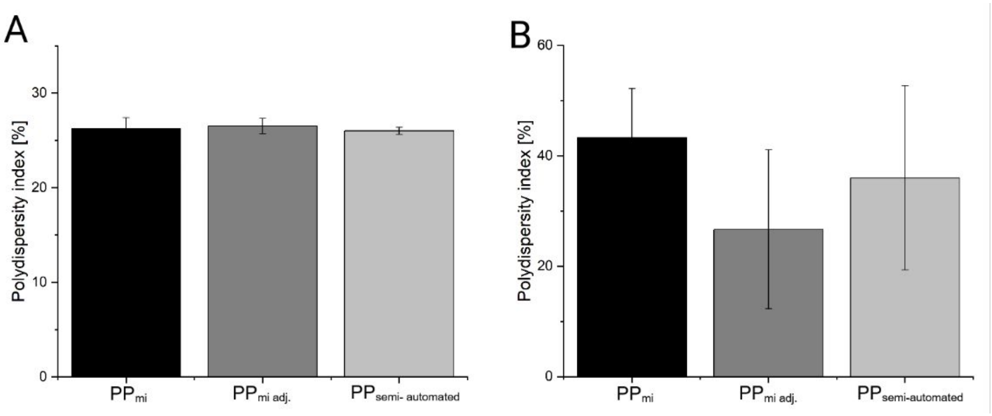

Figure S9. Comparison of polydispersity index of microfluidic-produced polyplexes at a N/P ratio 10. The polydispersity index of microfluidic-produced polyplexes were analyzed after complexation in HBG buffer (A) and after dilution in Opti-MEM (B). PP_{mi}: microfluidic-produced polyplexes in HBG; $PP_{mi\ adj.}$: microfluidic-produced polyplexes in HBG with adjusted start concentrations; $PP_{semi\text{-}automated}$: microfluidic-produced polyplexes in HBG followed by microfluidic-dilution with Opti-MEM, using adjusted start concentrations. Data represent mean ± SD with n=3. Statistical analysis was performed using Bonferroni correction: * $p \leq 0.05$; ** $p \leq 0.01$; *** $p \leq 0.001$.

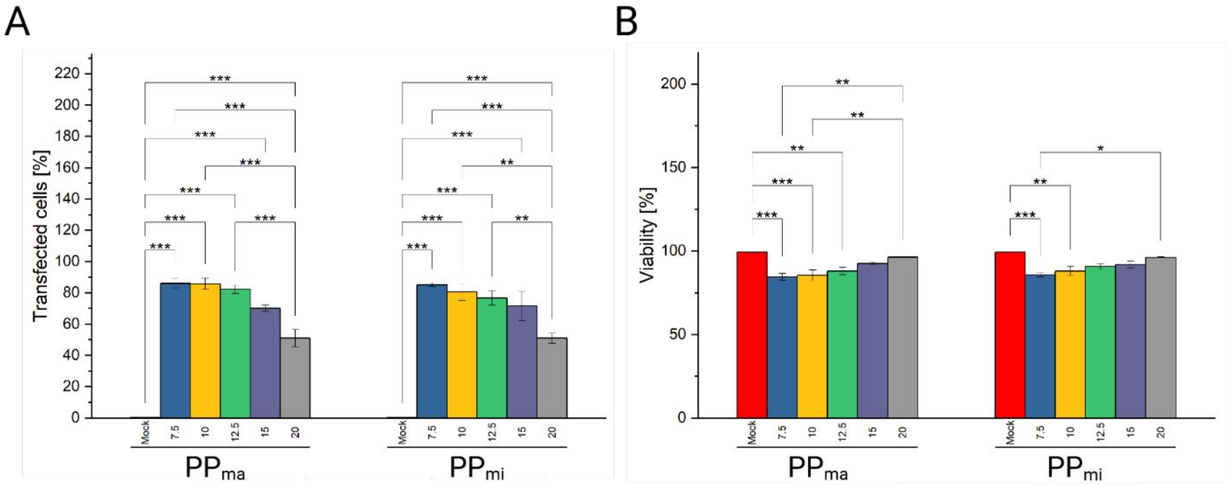

Figure S10. Statistical analysis for comparison of transfection efficiency (A) and cell viability (B) depending on manual and microfluidic (Setup B "mi") produced polyplexes and different N/P ratios. Total cells: 8×10^4 per well, 12-well plate, transfection volume 0.5 mL (0.05 mL polyplex solution), fix polymer density (40 µg polymer per 10^6 cells) and polymer concentration (6.4 µg/mL during transfection), N/P ratio adjusted by pDNA amount, recovery time post-transfection: 48 h. Data represent mean values ± SD with n=3. * p<=0.05; ** p<=0.01; *** p<=0.001.

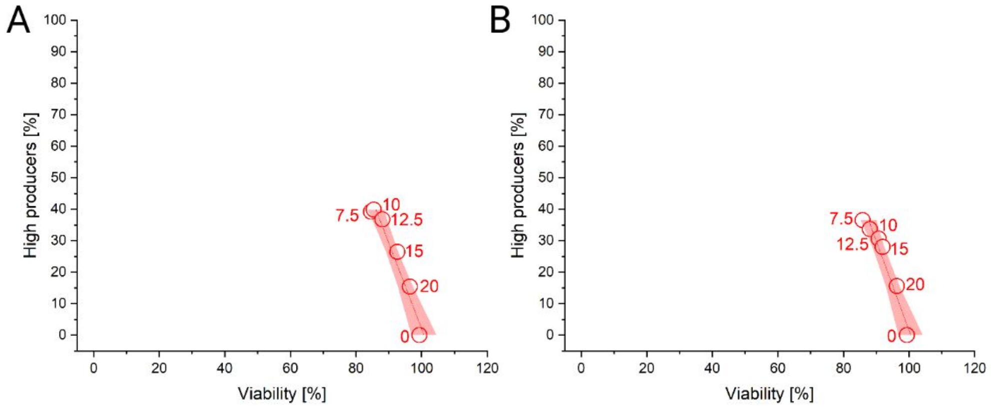

Figure S11. Relationship between the incidence of "high producers" and "viability" 48 h post-transfection, based on the data presented in Fig. 7.

A: Manual polyplexes production (Fig. 7, PP_{ma}), y= -0.37 x + 100.74 R^2: 0.927.

B: Microfluidic polyplexes production (Fig. 7, PP_{mi}), y= -0.35 x + 100.48 R^2: 0.908.

Red line: fit with 95 % confidence band.

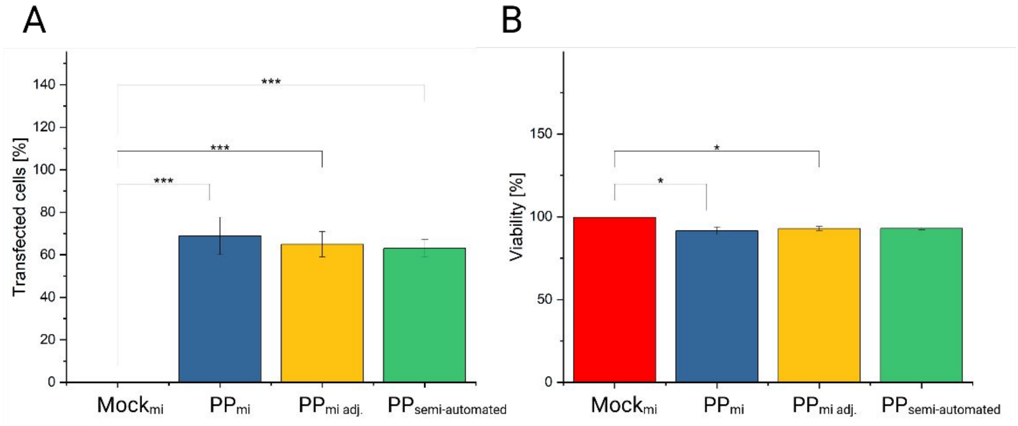

Figure S12. Statistical analysis for comparison of transfection efficiency (A) and cell viability (B) depending on different microfluidic produced polyplexes (Setup B "mi", "$mi_{adj.}$" and Setup C "semi-automated"). Total cells: 8 × 10^4 per well, 12-well plate, N/P ratio: 10, transfection volume 0.5 mL (0.05 mL polyplex solution), polymer density 40 µg per 10^6 cells, polymer concentration 6.4 µg/mL during transfection, contact time 2 h, recovery time post-transfection: 48 h. "PP_{mi}": Microfluidic-based complexation step (Setup B "mi") with manual-based dilution step; "$PP_{mi\ adj.}$": Microfluidic-based complexation step (Setup B "$mi_{adj.}$") with manual-based dilution step, using adjusted pDNA and PEI start concentrations; "$PP_{semi\text{-}automated}$" (Setup C "semi-automated"): Microfluidic-based complexation and dilution steps with adjusted pDNA and PEI start concentrations. Data represents mean values ± SD with n=3.
